# Hypermethylation-Mediated Silencing of *CIDEA*, *MAL* and *PCDH17* Tumour Suppressor Genes in Canine DLBCL: From Multi-Omics Analyses to Mechanistic Studies

**DOI:** 10.3390/ijms23074021

**Published:** 2022-04-05

**Authors:** Eleonora Zorzan, Ramy Elgendy, Giorgia Guerra, Silvia Da Ros, Maria Elena Gelain, Federico Bonsembiante, Giulia Garaffo, Nicoletta Vitale, Roberto Piva, Laura Marconato, Luca Aresu, Mauro Dacasto, Mery Giantin

**Affiliations:** 1 Department of Comparative Biomedicine and Food Science, University of Padua, 35020 Legnaro, Italy; eleonora.zorzan@gmail.com (E.Z.); ramy.elgendy@astrazeneca.com (R.E.); giorgiaguerra94@gmail.com (G.G.); silviadaros31@gmail.com (S.D.R.); mariaelena.gelain@unipd.it (M.E.G.); federico.bonsembiante@unipd.it (F.B.); mauro.dacasto@unipd.it (M.D.); 2 Discovery Biology, Discovery Sciences, R&D, AstraZeneca, 43150 Gothenburg, Sweden; 3 Department of Biomedical Sciences, Cedars-Sinai Medical Center, Los Angeles, CA 90048, USA; 4 Department of Animal Medicine, Productions and Health, University of Padua, 35020 Legnaro, Italy; 5 Molecular Biotechnology Center, Department of Molecular Biotechnology and Health Sciences, University of Turin, 10126 Turin, Italy; giulia.garaffo@gmail.com (G.G.); nicoletta.vitale@unito.it (N.V.); roberto.piva@unito.it (R.P.); 6 Department of Veterinary Medical Sciences, University of Bologna, 40064 Ozzano dell’Emilia, Italy; laura.marconato@unibo.it; 7 Department of Veterinary Sciences, University of Turin, 10095 Grugliasco, Italy; luca.aresu@unito.it

**Keywords:** diffuse large B-cell lymphoma, dog, tumour suppressor genes, DNA methylation, CiDEA, MAL, PCDH17

## Abstract

Gene expression is controlled by epigenetic deregulation, a hallmark of cancer. The DNA methylome of canine diffuse large B-cell lymphoma (cDLBCL), the most frequent malignancy of B-lymphocytes in dog, has recently been investigated, suggesting that aberrant hypermethylation of CpG loci is associated with gene silencing. Here, we used a multi-omics approach (DNA methylome, transcriptome and copy number variations) combined with functional in vitro assays, to identify putative tumour suppressor genes subjected to DNA methylation in cDLBCL. Using four cDLBCL primary cell cultures and CLBL-1 cells, we found that *CiDEA*, *MAL* and *PCDH17*, which were significantly suppressed in DLBCL samples, were hypermethylated and also responsive (at the DNA, mRNA and protein level) to pharmacological unmasking with hypomethylating drugs and histone deacetylase inhibitors. The regulatory mechanism underneath the methylation-dependent inhibition of those target genes expression was then investigated through luciferase and in vitro methylation assays. In the most responsive CpG-rich regions, an in silico analysis allowed the prediction of putative transcription factor binding sites influenced by DNA methylation. Interestingly, regulatory elements for *AP2, MZF1, NF-kB, PAX5* and *SP1* were commonly identified in all three genes. This study provides a foundation for characterisation and experimental validation of novel epigenetically-dysregulated pathways in cDLBCL.

## 1. Introduction

Diffuse large B-cell lymphoma (DLBCL) is the most aggressive malignancy of mature B-lymphocytes and accounts for approximately 50% of non-Hodgkin’s lymphomas in dogs [1]. Canine DLBCL (cDLBCL) exhibits many characteristics similar to the activated B-cell (ABC) form of human DLBCL, including the constitutive activation of the nuclear factor-kB (*NF-kB*) pathway, the double expression of *MYC* proto-oncogene (*MYC*), and *BCL2* apoptosis regulator, as well as the enrichment for genes in the *MYC* pathway [1,2,3].

Due to the variable clinical characteristics and treatment response of dogs with cDLBCL, many efforts have been made in the last 10 years to better characterise its molecular heterogeneity and understand the molecular mechanisms driving its pathogenesis. As a result, several -omics approaches were used to better profile this malignancy and consequently improve both its diagnosis and therapy. For instance, the transcriptome landscape of cDLBCL was characterised by microarrays [4,5,6] and RNA sequencing (RNA-seq) [7,8]. Other works focused on the copy number aberrations [7,9,10], the exome, and RNA mutational features [11,12]. To complete the cDLBCL molecular profile, the methylome [7,13] and the long noncoding RNA landscape [14] have also been recently investigated.

As a further improvement in the study of cDLBCL, Aresu and colleagues used a multi-layered genomics approach, where they integrated gene expression with copy number variations (CNVs) and genome-wide methylation data [7]. This integrative method allowed the identification of novel deregulated pathways and individual transcripts and provided insights to novel therapeutic targets. The multi-omics approach is considered a valuable tool for establishing the causal relationship between molecular signatures and the phenotypic manifestation of cancer hallmarks [15]. In particular, the integration of DNA methylation with RNA-seq is of value for studying the methylation regulation of genes [16].

DNA methylation occurs at the cytosine residues within cytosine–guanine sequences (CpG), often localized around promoter regions of genes. Historically, methylated CpG islands have been shown to inhibit gene expression by interfering with the binding of transcription factors (TFs), despite the fact that a more recent model proposes that DNA methylation and TF binding affect each other [17,18,19]. The epigenetic modification that impedes TF recruitment is crucial for tumourigenesis since it has been reported that the inactivation of certain tumour suppressor genes (TSGs) occurs as a consequence of hypermethylation within the promoter regions [20]. Previous studies have demonstrated the aberrant methylation and silencing of TSGs in canine B- and T-cell lymphoma cell lines [21,22,23,24] as well as in cDLBCL samples [25].

The identification of aberrantly methylated genes may provide a better understanding of cDLBCL pathogenesis [26], as well as pave the way for the development of novel tumour markers and therapeutic targets. Indeed, DNA methylation is reversible and, consequently, extremely interesting for therapeutic approaches incorporating hypomethylating drugs (HDs) and/or histone deacetylase inhibitors (HDACis), since they could reprogram cells rather than induce cytotoxicity, impact a variety of cellular processes at once, and potentiate the action of other therapeutic agents [27].

The aim of this study was to identify novel methylation-driven epigenetic alterations in cDLBCL and, consequently, investigate their regulatory mechanisms. To achieve that, we performed a genome-wide screening of the aberrantly methylated CpG islands and the associated down-regulated genes in cDLBCL. Following a series of in vitro and in silico experiments, we selected three candidate genes, characterised their DNA methylation and gene expression status in cDLBCL primary cell cultures (PCCs) and CLBL-1 cells, performed mechanistic studies and unveiled the putative transcription factor binding sites (TFBSs) involved in their methylation-dependent transcription inactivation. Those genes are namely cell-death-inducing DNA fragmentation factor α-like effector A (*CiDEA*), Myelin and Lymphocyte protein (*MAL*) and Protocadherin 17 (*PCDH17*), and we describe our study in more detail herein.

## 2. Results

### 2.1. Identification of Putative TSGs Deregulated in cDLBCL

**Figure 1:**
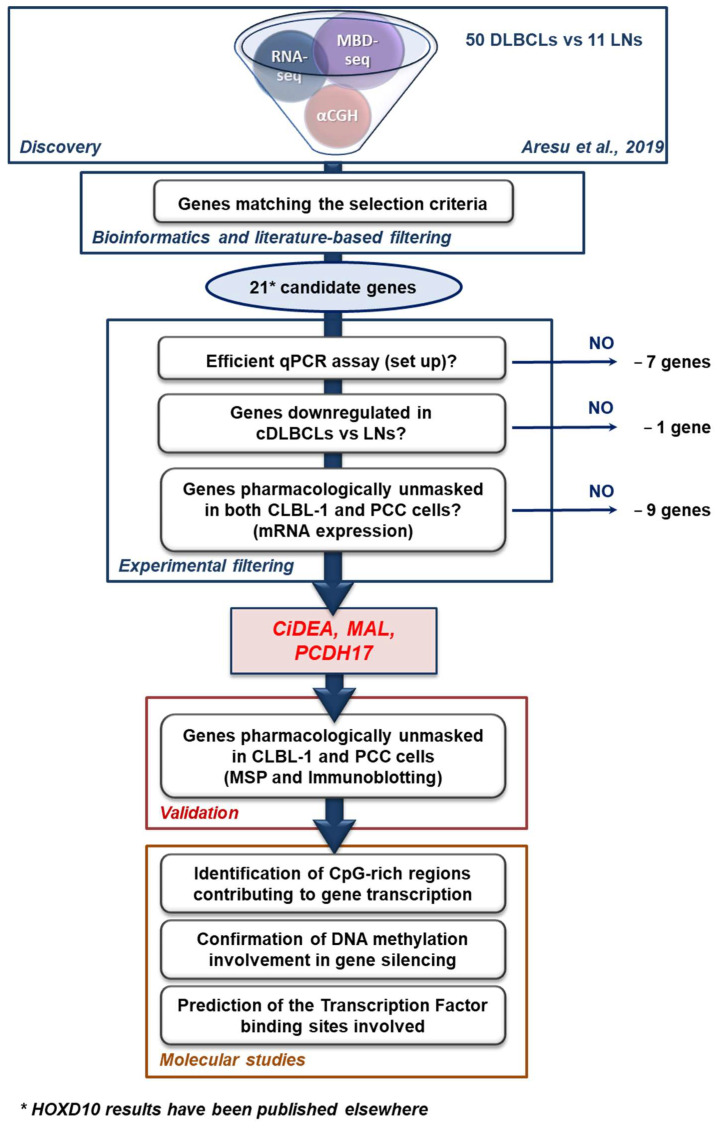
Flow chart of the study.

A flow chart summing up the study design is reported in Figure 1.

The genomic profiling of 50 cDLBCL samples and 11 control lymph nodes (LNs) was previously performed using RNA-seq, Methyl-CpG-binding (MBD) sequencing, and Array Comparative Genomic hybridization (αCGH) approaches [7]. Gene expression, DNA methylation, and CNVs data of tumour and non-tumour tissues (published in [7]) were integrated to obtain the first set of not redundant protein-coding genes (*n* = 309) that simultaneously encompassed the following features in cDLBCL specimens: (1) significant hypermethylation in promoter and/or intergenic and/or exonic regions; (2) significant downregulation or complete silencing; (3) CNVs-free (gain or losses) (lists 1–4, Figure S1). A further list of 30 genes (list 5, Figure S1), represented by not redundant hypermethylated, downregulated and CNVs-free genes, and showing a significant (*p* < 0.05) inverse correlation between DNA methylation (MET) and gene expression (GEX) data, complemented the first four lists of genes (Figure S1). As a consequence, an overall set of 339 candidate genes was initially defined. The list was reduced first to 50 hits using a literature-based filtering approach and finally to 21 (Figure S1) using the selection criteria described in Materials and Methods. Overall, the combined bioinformatics and literature-based screenings allowed the selection of 21 putative TSGs, considered in the subsequent experimental filtering (Figure 1). Among them, *HOXD10* was previously identified and validated by the same research group [13,24]; further results related to *HOXD10* will be published elsewhere.

**Figure 2:**
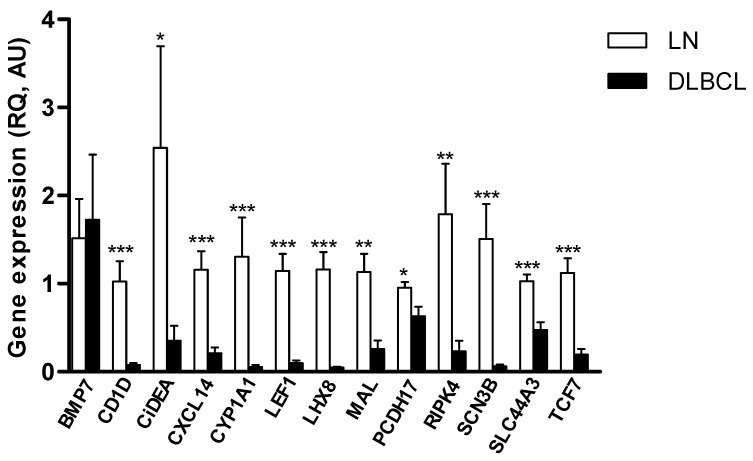
Gene expression of 13 candidate TSGs in cDLBCLs and control LNs. The mRNA expression was evaluated by qPCR in 12 cDLBCL and 10 LN samples. The relative expression values (RQ) are expressed in arbitrary units (AU), as the mean ± SEM. Statistical analysis: Mann Whitney test. *: *p* < 0.05; **: *p* < 0.01; ***: *p* < 0.001.

The number of target genes was reduced to 13 units after the qPCR assay set up, as 7 qPCR assays (*AJAP1, BCL11B, CLDN3, HOXA11, PAK5, PCDH10, TEKT3*) did not match the efficiency parameters considered acceptable for samples analysis (90% < E < 110%), due to the low mRNA expression in the lymphoid tissue or to the presence of multiple splice variants. As a consequence, the gene expression analysis of tumour and control samples was performed for the following genes only: *BMP7, CD1D, CiDEA, CXCL14, CYP1A1, LEF1, LHX8, MAL, PCDH17, RIPK4, SCN3B, SLC44A3* and *TCF7*. The overall results are reported in Figure 2. All target genes, except *BMP7*, were significantly downregulated in cDLBCL specimens (*p* < 0.05) and were taken into consideration in the following steps of the study.

### 2.2. CiDEA, MAL and PCDH17 mRNA Expression Is Restored by HDs and HDACis in Both CLBL-1 Cells and cDLBCL Primary Cell Cultures

**Figure 3:**
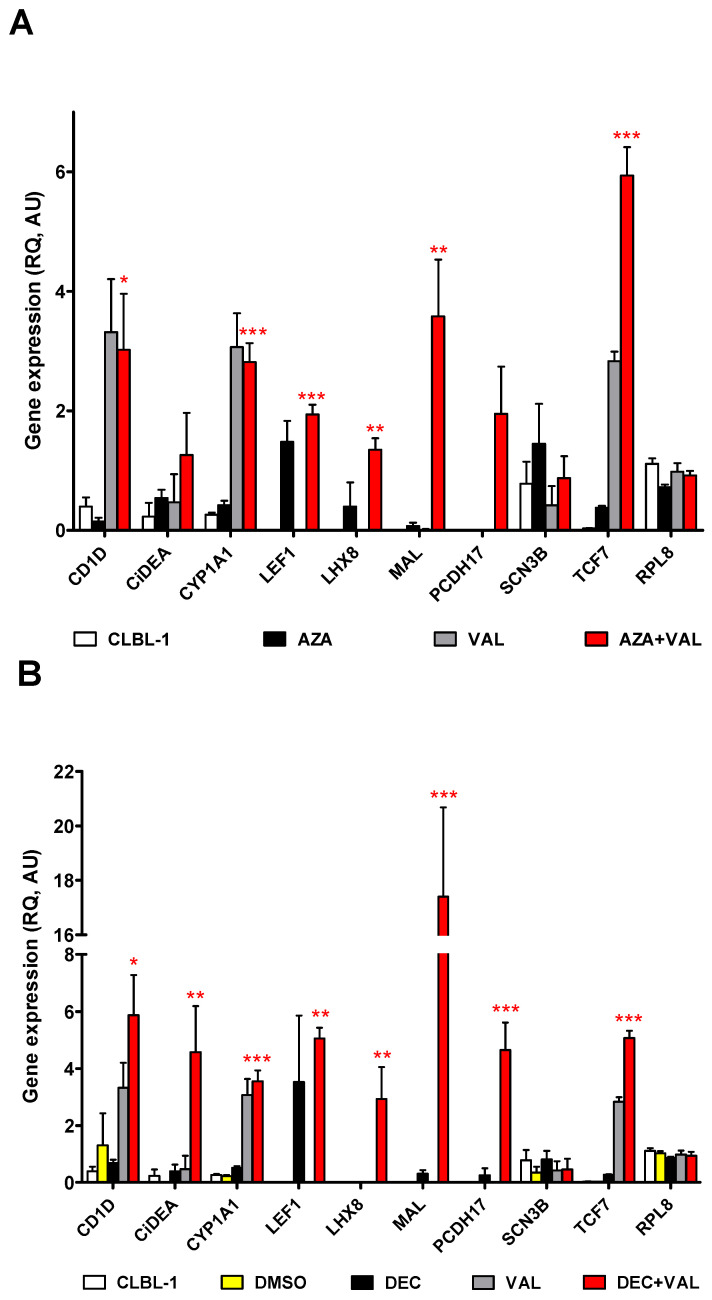
Effect of the treatment with AZA or DEC in combination with VAL on the mRNA expression of 9 candidate TSGs in CLBL-1 cells. (**A**) AZA, (**B**) DEC. The expression levels of the target mRNAs (relative expression values, RQ), evaluated by qPCR and normalized to *GOLGA1* and *CCZ1*, are expressed in arbitrary units (AU), as the mean ± SEM of four independent experiments. *CXCL14, RIPK4* and *SLC44A3* are not shown because they were not expressed in CLBL-1 cells both in the control and treatment conditions. *RPL8*, the negative control gene, was not affected by the treatment, as expected. Statistical analysis: ANOVA + Bonferroni post hoc test. *: *p* < 0.05; **: *p* < 0.01; ***: *p* < 0.001. The statistical significance between CLBL-1 vs. AZA + VAL and DMSO vs. DEC + VAL only is shown.

**Figure 4:**
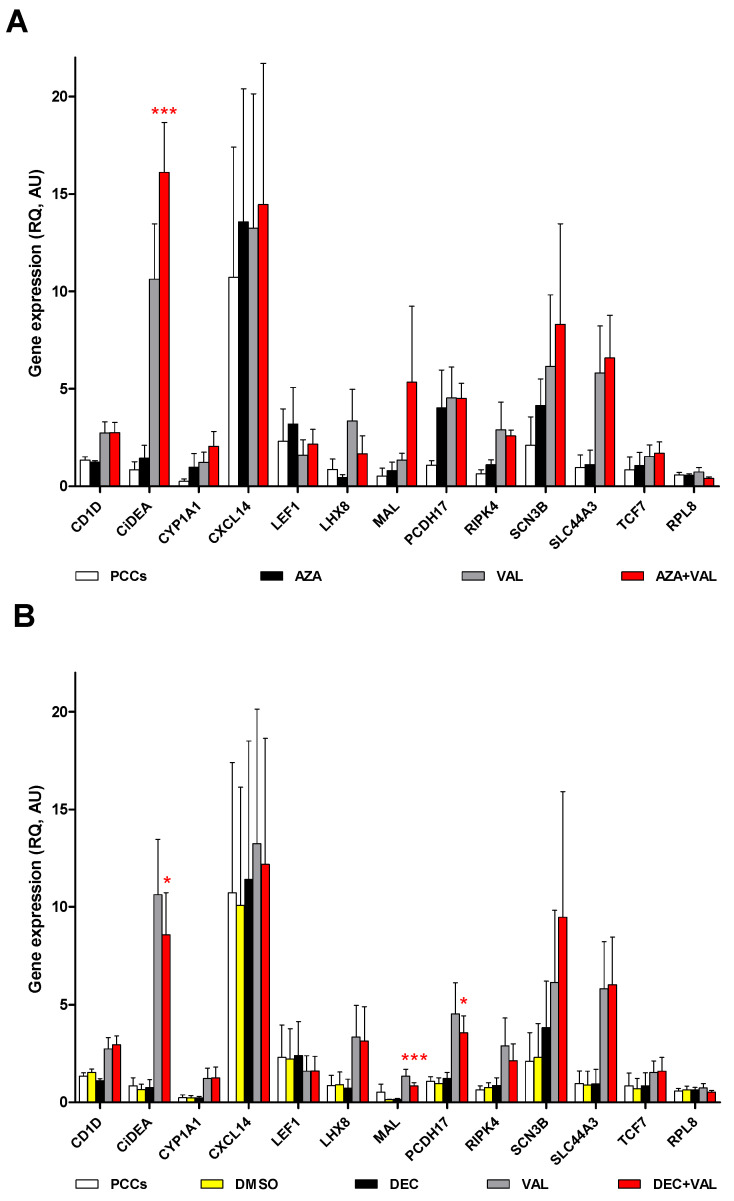
Effect of the treatment with AZA or DEC in combination with VAL on the mRNA expression of 12 candidate TSGs in four PCCs. (**A**) AZA, (**B**) DEC. The expression levels of the target mRNAs (relative expression values, RQ), evaluated by qPCR and normalized to *GOLGA1* and *CCZ1*, are expressed in arbitrary units (AU), as the mean ± SEM. *RPL8*, the negative control gene, was not affected by the treatment, as expected. Statistical analysis: ANOVA + Bonferroni post hoc test. *: *p* < 0.05; ***: *p* < 0.001. The statistical significance between PCCs vs. AZA + VAL and DMSO vs. DEC + VAL only is shown.

To confirm the DNA methylation-induced silencing of the 12 candidate genes previously identified, pharmacologically re-expression experiments in CLBL-1 and four cDLBCL PCCs were conducted. Cells were treated with HDs and HDACis either alone or in combination. The main gene expression results, obtained with valproic acid (VAL), are reported in Figure 3 and Figure 4; those deriving from the experiments with vorinostat (SAHA) and trichostatin A (TSA) are presented in Figures S2 and S3.

In CLBL-1 cells, azacytidine (AZA) and decitabine (DEC) alone showed a mild effect on gene re-expression, while the co-treatment with the HDACis magnified the effects, as expected; AZA + VAL and/or DEC + VAL significantly restored (*p* < 0.05) the mRNA expression of eight out of nine genes (Figure 3). Specifically, comparable results were obtained for *CD1D, CYP1A1, LEF1, LHX8, MAL* and *TCF7* with AZA + VAL (Figure 3A) and DEC + VAL (Figure 3B) treatments; however, *CiDEA* and *MAL* were significantly affected when DEC only was used as HD (*p* < 0.01 and *p* < 0.001, respectively). *CXCL14, RIPK4* and *SLC44A3* were not detectable in CLBL-1 cells both in control and treatment conditions, while *SCN3B* and the negative control gene *RPL8* did not show any effects. The SAHA and TSA combinations allowed the re-expression of a comparatively lower number of genes (*n* = 6), with less net and significant results (Figure S2).

The effect of HDs and HDACis was also tested in four PCCs using the same experimental protocol and the drug concentrations chosen for CLBL-1 cells. When compared to CLBL-1 cells, characterised by one clone only, PCCs were more heterogeneous in terms of cell composition, with a 69.3% mean percentage of B-cells (Table S1); these cells were characterised by a higher sensitivity to VAL 1.7 mM (cytotoxicity > 20%) and a lower sensitivity to DEC 0.13 µM (cytotoxicity < 10%: Table S2). Overall, PCCs expressed all the target genes and showed a highly variable response to the treatments (Figure 4); in particular, the addition of AZA + VAL (Figure 4A) and/or DEC + VAL (Figure 4B) significantly restored only the mRNA expression of *CiDEA, MAL* and *PCDH17* (*p* < 0.05). Similar results were obtained for *CiDEA* when the combination AZA + TSA was used (Figure S3). As a whole, *CiDEA, MAL* and *PCDH17* were the sole genes consistently unmasked by epigenetic drugs both in CLBL-1 and PCC cells (5/5 cell lines); consequently, they were identified as the most likely candidates for DNA methylation-induced silencing in cDLBCL and were selected as the definite target genes of the present study.

### 2.3. CiDEA, MAL and PCDH17 Are Confirmed to Be Aberrantly Hypermethylated in CLBL-1 and PCC Cells

**Figure 5:**
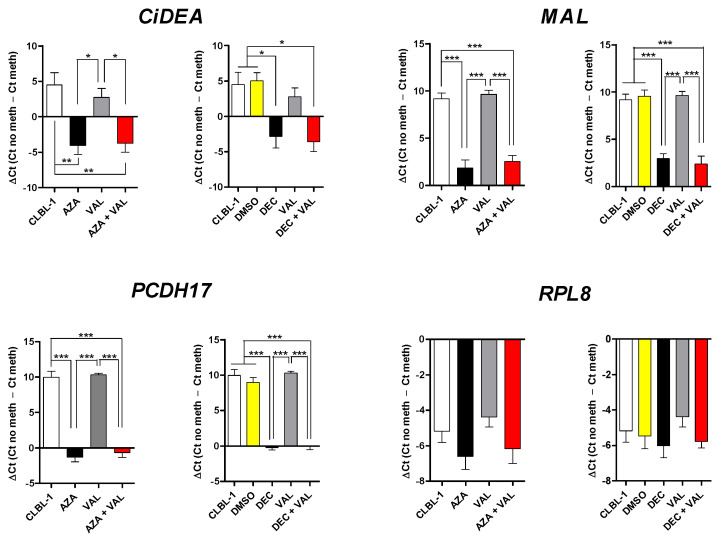
Effect of the treatment with AZA or DEC in combination with VAL on *CiDEA, MAL*, *PCDH17* and *RPL8* methylation status in CLBL-1 cells. For each gene, the results of AZA are in the graph on the left, while the results of DEC are in the graph on the right. The results of MSP analyses (qPCR) are expressed as ∆Ct (=Ct No meth − Ct meth), as the mean ± SEM of four independent experiments. Statistical analysis: ANOVA + Bonferroni post hoc test. (*: *p* < 0.05; **: *p* < 0.01; ***: *p* < 0.001). *RPL8* (the negative control gene) was not affected by the treatment as expected.

**Figure 6:**
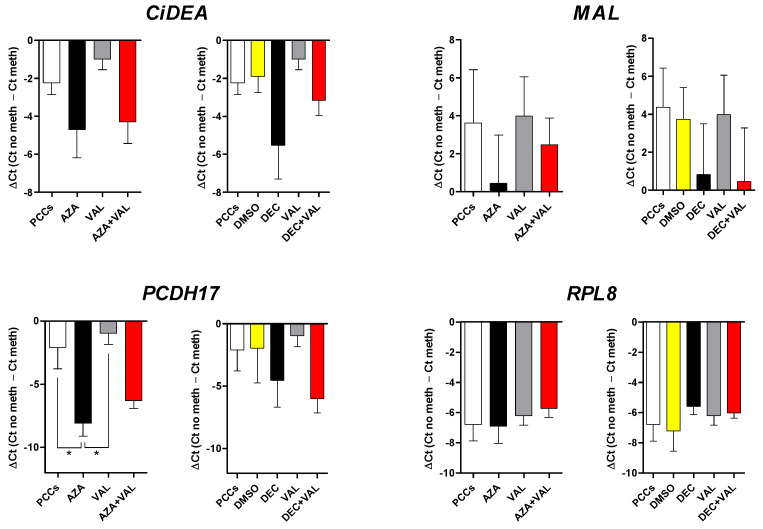
Effect of the treatment with AZA or DEC in combination with VAL on *CiDEA, MAL, PCDH17* and *RPL8* methylation status in PCC cells. For each gene, the results of AZA are in the graph on the left, while the results of DEC are in the graph on the right. The results of MSP analyses (qPCR) are expressed as ∆Ct (=Ct No meth − Ct meth) as the mean ± SEM. Four independent PCCs were analysed. Statistical analysis: ANOVA + Bonferroni post hoc test (*: *p* < 0.05). *RPL8* (the negative control gene) was not affected by the treatment as expected.

The methylation status of one representative CpG-rich region for *CiDEA, MAL* and *PCDH17* was evaluated by Methyl Specific PCR (MSP) in the control and treated CLBL-1 and PCC cells. The results deriving from the association of AZA/DEC + VAL are shown in Figure 5 and Figure 6, while those regarding the use of SAHA and TSA in combination with the two HDs are reported in Figures S4 and S5.

In CLBL-1 cells, the treatment with HDs (AZA or DEC) alone or in combination with VAL significantly decreased the methylation of the three genes under analysis (Figure 5; *p* < 0.05 for *CiDEA*, *p* < 0.001 for *MAL* and *PCDH17*), while VAL alone was ineffective, as expected. Consistently with VAL, SAHA and TSA alone or in association with HDs showed comparable results (Figure S4). An overall decrease of *CiDEA, MAL* and *PCDH17* methylation profile, albeit not significant, was also observed in PCCs after the treatment with HDs alone or in association with HDACis (Figure 6 and Figure S5). Because of the high variability recorded among the four PCCs, statistically significant differences were obtained only for *PCDH17* in PCCs vs. AZA (*p* < 0.05: Figure 6). In both CLBL-1 and PCC cells the methylation of *RPL8*, the negative control gene, was never significantly affected by the treatments. As a whole, these results confirmed the aberrant hypermethylation of *CiDEA, MAL* and *PCDH17* CpG sites in CLBL-1 and PCC cells.

### 2.4. CiDEA and MAL Protein Expression Is Restored by AZA + VAL in CLBL-1 Cells

**Figure 7:**
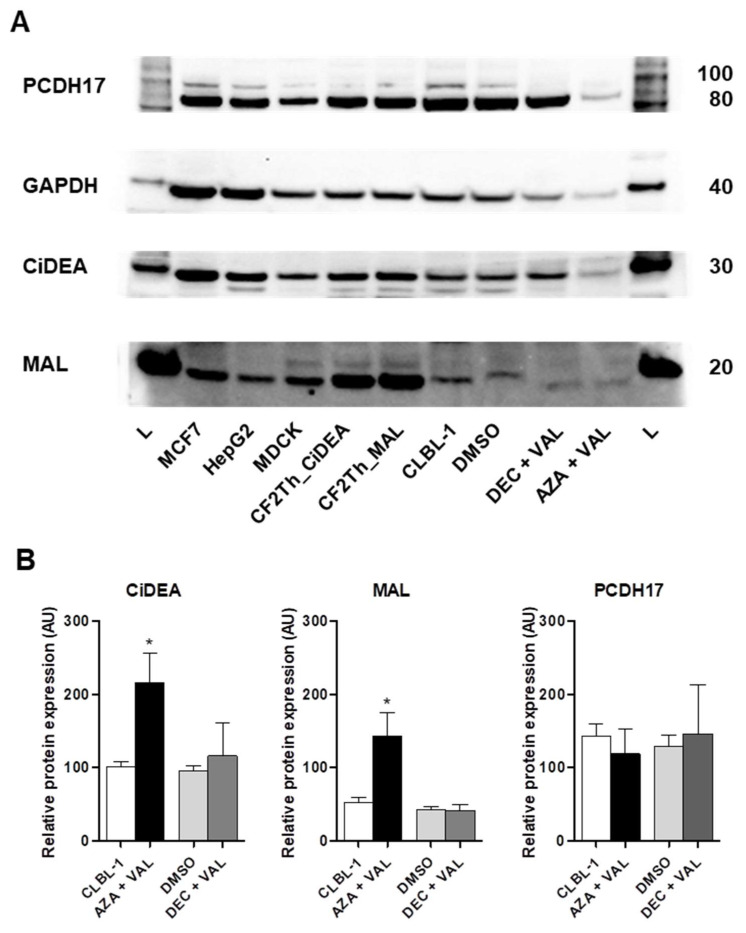
Effect of AZA + VAL or DEC + VAL combinations on CiDEA, MAL and PCDH17 protein expression in CLBL-1 cells. (**A**) Whole protein lysates from treated and untreated cells were subjected to immunoblotting, using GAPDH as the loading control. The image is representative of six independent experiments (independent cell cultures). MCF7, HepG2 and MDCK cell lines as well as CF2Th cells transfected with canine *CiDEA* and *MAL* full sequences have been used as positive controls (human and canine). Thirty and ~15 µg of total proteins were loaded in each well for the positive controls and CLBL-1 cells, respectively. (**B**) For the protein quantification of each sample, the integrated optical density (IOD) of the specific bands was normalized first to the corresponding GAPDH IOD and subsequently to the canine positive control, selected as the calibrator (CF2Th_CiDEA for CiDEA, CF2Th_MAL for MAL and MDCK for PCDH17). The results of the densitometric analysis are expressed in arbitrary units (AU) as the mean ± SEM of six independent experiments (independent cell cultures). Statistical analysis: Mann Whitney U-test (*: *p* < 0.05). L: ladder.

To evaluate if the pharmacological unmasking of *CiDEA, MAL* and *PCDH17* CpG-rich regions was associated to a restoration of the expression of the respective proteins, immunoblotting analyses (Figure 7A) were performed in CLBL-1 cells treated with HDs in combination with VAL, the most effective HDACi tested. This same approach was not applied in PCC cells due to the limited number of available cells. The overall results are reported in Figure 7B. The protein expression of CiDEA and MAL was significantly induced (~2- or 3-fold, *p* < 0.05) only when VAL was used in combination with AZA. PCDH17 protein expression was never affected by the treatments.

### 2.5. In Vitro Methylation of CiDEA, MAL and PCDH17 CpG Sites Affects Gene Transcription

To assess the role of DNA methylation on the regulation of the target genes, two subsequent sets of in vitro studies were performed.

**Figure 8:**
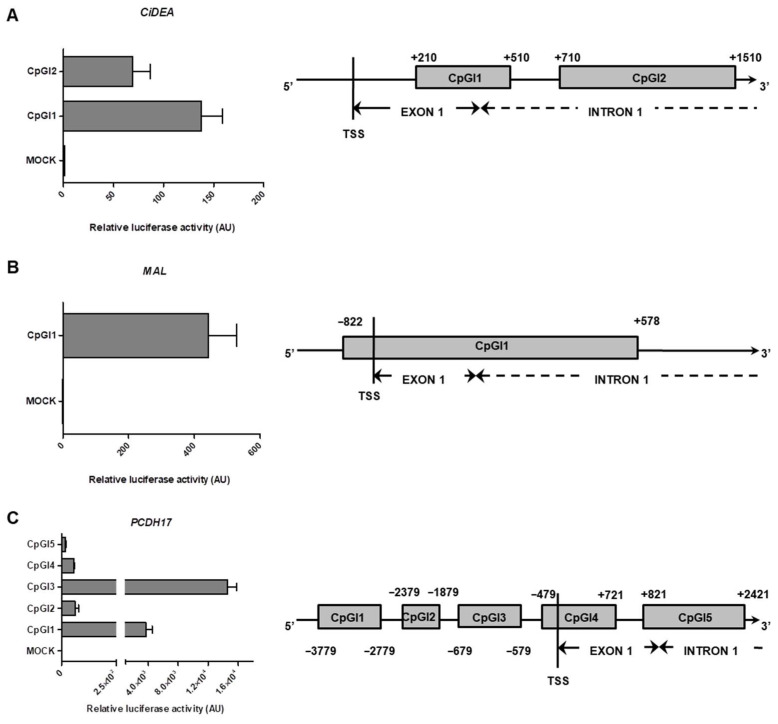
Luciferase assays of pCpGL-basic constructs containing *CiDEA* (**A**), *MAL* (**B**) and *PCDH17* (**C**) CpG islands (CpGI) in CF2Th transfected cells. Luciferase activity values are expressed in arbitrary units (AU) as the fold activation relative to pCpGL-basic-mock-transfected cells (mean ± SEM of three independent experiments). On the right, a schematic diagram of *CiDEA*, *MAL* and *PCDH17* gene structure and CpG-rich region position respect to the transcription starting site (TSS) is reported.

The first set of experiments aimed to screen cloned CpG-rich regions (see Figure 8 for details) and evaluate if they possessed necessary and sufficient regulatory elements for the activation of gene transcription. To this purpose, the luciferase activity of the unmethylated plasmids was preliminarily assayed. Two CpG-rich sequences (namely CiDEA_CpGI1 and CiDEA_CpGI2) were considered for *CiDEA* (Figure 8A); both of them located downstream to the transcription starting site (TSS; exon 1 and intron 1), showed a significant increase of the luciferase signal (140- and 70-fold, respectively) with respect to the empty vector. A single cloned *MAL* region, overlapping the TSS (i.e., MAL_CpGI1) and 1400 bp long (Figure 8B) activated the luciferase transcription 400 times more than the control. Concerning *PCDH17*, the luciferase activity was tested in five constructs, namely PCDH17_CpGI1, CpGI2, CpGI3, CpGI4, CpGI5 (three upstream, one overlapping and one downstream to the TSS, Figure 8C). All of them induced the transcription of the reporter gene; nevertheless, the PCDH17_CpGI1 and PCDH17_CpGI3 constructs were the most effective ones, showing a luciferase signal 3500- and 14,500 times higher than control, respectively. Considering the results of this preliminary screening as a whole, CiDEA_CpGI1, MAL_CpGI1, PCDH17_CpGI1 and PCDH17_CpGI3 were the CpG-rich regions that exhibited the highest transcription activation of the respective promoter and were selected for the subsequent in vitro methylation assays.

**Figure 9:**
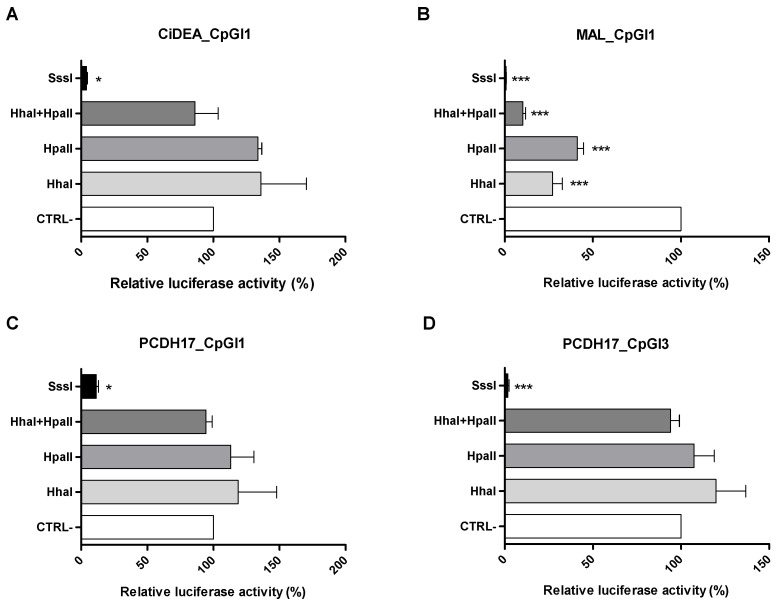
Luciferase activity of in vitro methylated CiDEA_CpGI1 (**A**), MAL_CpGI1 (**B**), PCDH17_CpGI1 (**C**) and PCDH17_CpGI3 (**D**) plasmids in CF2Th cells. Plasmids containing the CpG sites mostly involved in the regulation of *CiDEA*, *MAL* and *PCDH17* transcription were subjected to in vitro methylation. SssI, HhaI and HpaII methylation enzymes were used separately or in combination (HhaI + HpaII). Luciferase activity values (mean ± SEM of three independent experiments) are expressed as the percentage of the negative control (CTRL-, the respective unmethylated plasmid) activity, to which an arbitrary value of 100% was assigned. Statistical analysis: ANOVA + Bonferroni post hoc test (*: *p* < 0.05; ***: *p* < 0.001).

To prove if the methylation could affect the transcription, the selected plasmids were methylated using SssI, HhaI and HpaII methyltransferases and subsequently tested through luciferase assays. The results are shown in Figure 9. The maximum inhibition of the luciferase transcription was obtained with the whole methylation using the SssI enzyme (*p* < 0.05 for CiDEA_CpGI1 and PCDH17_CpGI1, *p* < 0.01 for MAL_CpGI1 and PCDH17_CpGI3). Indeed, an average decrease of about 95% was obtained in all four SssI-methylated plasmids compared to the respective unmethylated clones. As regards HhaI and HpaII enzymes, they did not show any significant effect on CiDEA_CpGI1, PCDH17_CpGI1 and PCDH17_CpGI3; conversely, they significantly decreased (72% and 59%, respectively) the luciferase signal in MAL_CpGI1 (*p* < 0.001). The effect of the partial methylation was even magnified when the two methyltransferases were used in combination; as a matter of fact, in cells transfected with the HhaI + HpaII-methylated plasmid, a residual luciferase activity of about 10% with respect to that of the unmethylated plasmid was observed. As a whole, the results here obtained suggested that the methylation of the cis-regulatory CpG sites within the fragments CiDEA_CpGI1, MAL_CpGI1, PCDH17_CpGI1 and PCDH17_CpGI3 most likely determined the mRNA expression level of *CiDEA, MAL* and *PCDH17*, respectively.

### 2.6. Transcription Factor Binding Sites Putatively Iinvolved in CiDEA, MAL and PCDH17 Methylation-Dependent Silencing

**Figure 10:**
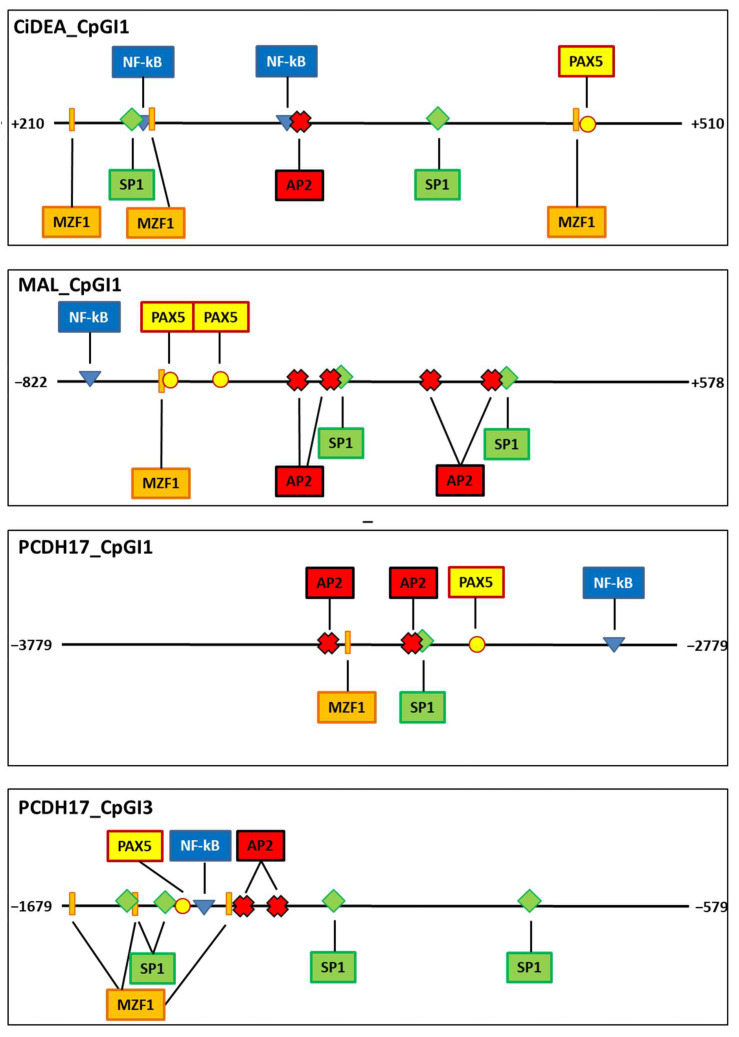
Graphical representation of CiDEA_CpGI1, MAL_CpGI1, PCDH17_CpGI1 and PCDH17_CpGI3 regions showing the putative localisation of *AP2, MZF1, NF-kB, PAX5* and *SP1* TFBSs predicted at SssI, HhaI and HapII methylation sites using MatInspector, Promo3 and TFbind tools. In CiDEA’s graph the position of the TATA box is also shown.

From an in silico analysis of the four regions that underwent methylation studies, several putative TF motifs containing CpG dinucleotides were identified at SssI, HhaI and HapII methylation sites or in strict proximity. Specifically for *MAL*, the highest priority was given to the TFBSs recognized by HhaI and HapII, based on the in vitro methylation results described above. Overall, we focused on the predicted TFBSs that, on a literature basis, were subjected to methylation and/or played a pivotal role in gene transcription in lymphoma or other neoplasms. A comprehensive outline of the results is reported in Table S3. Interestingly, TFBSs for *AP2, MZF1, NF-kB, PAX5* and *SP1* were commonly identified in all three genes (four regions out of four). A graphical representation of CiDEA_CpGI1, MAL_CpGI1 and PCDH17_CpGI1 and PCDH17_CpGI3 regions, showing the position of the main predicted TFBSs, is reported in Figure 10.

## 3. Discussion

This study aimed at identifying novel driver genes in cDLBCL and explored the role of methylation in the suppression of those genes.

The use of a multi-omics approach (DNA methylation, RNA-seq and αCGH) coupled with bioinformatics and literature-based screening allowed the detection of 21 putative TSGs with low expression profiles, hypermethylation status in the promoter region and no chromosome loss. One of these genes was *HOXD10*, a gene previously identified and validated in cDLBCL [13,24].

Following a series of pharmacological re-expression experiments, we identified three genes (*CiDEA, MAL* and *PCDH17*) that were either silenced or slightly expressed in cDLBCL and positively responded to the treatment with HDs and HDACis in either CLBL-1 or four cDLBCL PCCs. On the one hand, the findings were quite significant and easy to interpret in the CLBL-1 cells since they are composed of a single cellular component (B cells) [28]. On the other hand, PCCs showed wide variability in the response because of their high heterogeneity in terms of cell composition (both B- and T-cells) and a lower sensitivity toward DEC. However, despite this heterogeneity, the statistically significant outcomes in the PCCs indicate that *CiDEA*, *MAL* and *PCDH17* are appropriate choice as candidate genes.

*CiDEA* is a member of the cell-death-inducing DFF45 (DNA fragmentation factor-45)-like effector (CIDE) family [29]. It is considered a pro-apoptotic factor since it induces cell death associated with DNA fragmentation [29,30,31] and is frequently down-regulated in multiple human carcinomas [32,33]. It is considered a TSG as it regulates oesophageal squamous cell carcinoma proliferation and apoptosis through the JNK p21/Bad pathway and, when overexpressed, it causes decreased cell growth, foci formation and DNA replication in cells, as well as decreased tumourigenesis in nude mice [33].

*MAL* is a T-cell differentiation protein and an essential component of glycolipid-enriched membrane micro-domains or rafts involved in the apical transport of membrane and secretory proteins [34]. It is implicated in carcinogenesis in two opposite ways, as a tumour suppressor or tumour progression factor, based on the proteins, with specific functional roles, interacting with it [35]. The first evidence of its tumour suppressor capability were described in [36,37]; *MAL* ectopic expression either reduced tumour growth in nude mice or diminished cell motility, blocked G1/S transition and increased the Fas-mediated apoptosis in vitro [36]. In addition, *MAL* acts as a tumour progression factor in some kinds of lymphoma [35]. Specifically, *MAL* overexpression allowed the differentiation of acute from chronic adult T-cell leukaemia/lymphoma [38], and primary mediastinal large B-cell lymphoma [39,40] from DLBCL, in which it is sporadically expressed [41]. *MAL* is frequently silenced and hypermethylated in multiple human malignancies [36,37,42,43,44,45,46,47].

The *PCDH17* gene, encoding for protocadherin 17, belongs to the superfamily of protocadherins. These proteins play important roles in the regulation of cell adhesion and signalling transduction [48]; therefore, the repression of their expression might contribute to tumourigenesis. *PCDH17* has been recently defined as a new methylation driver gene that plays a critical role in the initiation, promotion and progression of different human tumours [16]. *PCDH17* is frequently downregulated and meantime hypermethylated in various human carcinomas [48,49,50,51]; it is considered a TSG [48,52,53,54,55], and its biological function in tumour pathogenesis was discovered at first in breast tumour cell lines [54]. The restoration of its expression through ectopic expression caused cell proliferation and mobility inhibition, cell cycle arrest and apoptosis, as well as the decreased expression of active β-catenin and its downstream target genes cyclin D1 and *MYC*; moreover, it reversed epithelial mesenchymal transition [54].

In general, the published data on *CiDEA, MAL* and *PCDH17* is largely from human oncology; thus, and to the best of the authors’ knowledge, they have never been investigated in dogs. The current findings show that (a) methylation-dependent silencing of these genes may occur in cDLBCL; and (b) this mechanism may influence cDLBCL development. Nonetheless, future functional studies are planned to depict the potential role of these genes in the pathogenesis and progression of cDLBCL.

Despite the fact that the pharmacological unmasking of *CiDEA, MAL* and *PCDH17* genes in CLBL-1 cells was consistent with the influence on DNA methylation status and protein expression, several exceptions were observed. While the demethylation of particular loci for all three genes was validated, the restoration of protein expression was only found for *CiDEA* and *MAL* when the AZA + VAL combination was utilized. This distinct effects of AZA and DEC combination on protein expression might be due to the previously reported sensitivities of CLBL-1 cells to those drugs [24] or to the differing mechanisms of action of the two cytidine analogues [56]. Furthermore, a prior investigation in T-cell lymphoma cell lines found various differentially expressed genes and differently regulated pathways depending on the HD employed, lending credence to the idea that DNMT inhibitors have gene-specific effects [24,57]. The lack of PCDH17 protein re-expression after AZA + VAL treatment, on the other hand, could be attributed to several factors, including: (a) the low specificity of the primary antibody against the canine protein; (b) the length of the treatment and/or the dose used, which were potentially insufficient for PCDH17 protein re-expression; and (c) the presence of post-transcriptional regulatory mechanisms. In this respect, epigenetic modulation of miR-196b, an oncogenic miRNA discovered in many human malignancies [58,59,60] and targeting *PCDH17* mRNA [61] was seen in human leukaemia cells [62]. As a result, we suggest that the use of epigenetic compounds (i.e., AZA/VAL) activated some post-transcriptional regulatory mechanisms of the PCDH17 protein, neutralizing the expected direct effect of HDs and HDACis on PCDH17 re-expression.

Overall, both CLBL-1 and PCC cells demonstrated clear effects of AZA and DEC on *CiDEA, MAL* and *PCDH17* methylation profiles (MSP), while mRNA re-expression was very poor when AZA or DEC were used alone. However, gene re-expression was consistent and significant when the HD was combined with VAL, SAHA or TSA. These findings suggest that a longer incubation time or a more effective DNMT inhibitor could be used [63], or that the expression of each gene may be partially regulated by other mechanisms such as histone deacetylation, as previously discovered [44,55,64].

Among the HDACis chosen in the present study, VAL showed the most consistent effects on gene expression restoration; thus, it was selected as unique HDACi to be used in association with AZA and DEC for protein investigations (immunoblotting analyses). Nevertheless, it should be considered that this compound recently showed some limitations; apart from the well-known inhibition of histone deacetylases, it might also exert direct immunomodulatory effects by interfering with the lymphocytes’ activating signalling pathways; in particular, it might reduce cell activation through protein kinase C inhibition [65].

Because methylation-induced gene repression may be influenced, at least in part, by methylation of TF binding sites [17,18,19], we investigated the regulatory mechanism underlying methylation-dependent inhibition of *CiDEA, MAL* and *PCDH17* transcription. Starting with genome-wide methylation data, we identified the most important CpG-rich regions of each gene and identified the regions primarily responsible for transcription activation using luciferase gene assays; we then confirmed the inhibitory effect of in vitro methylation on luciferase activation and predicted the main TFBSs subjected to methylation and potentially involved in gene silencing.

*MAL* and *PCDH17* CpG-rich regions have never been functionally characterised in humans or canines, and, to the best of the authors’ knowledge, the only available data concern the localisation and methylation profile of specific CpGIs in human cancers [37,42,66,67]. This means that the significance of CpG sites in the *MAL* and *PCDH17* promoter regions was merely inferred from the negative association between the methylation state of certain CpGIs and gene expression. *CiDEA* promoter, on the other hand, has been partially characterized in human fat cells [68] and murine liver cells [69]. Both human and murine *CiDEA* promoters contain common evolutionarily conserved regions that overlap with CpGIs, and candidate TFBSs for *SP1* and *NF-kB* have been proposed [68]. *SP1/SP3* binding sites were shown to be required for *CiDEA* promoter activity; moreover, methylation of CpG sites within these regions reduced transcription [70]. The TATA box and many putative TFBSs for *SP1* and *NF-kB* were discovered in the current study’s predictive analysis on *CiDEA* CpGI1, indicating a shared mechanism of *CiDEA* regulation across humans and dogs that require further investigation.

The in silico analysis of the CpG-rich areas that were most sensitive to in vitro methylation allowed us to identify five candidate TFBSs (*AP2, MZF1, NF-kB, PAX5* and *SP1*) that were present in multiple copies in all three genes and four CGIs. This implies that these TFs may play an important role in the transcriptional control of the TSGs under investigation and that their binding affinity to DNA motifs may be modified by CpG methylation state. A recent article in human chronic lymphocytic leukaemia (CLL) found that regulatory elements acquired and lost as a result of epigenetic modifications were enriched for the binding sites of the well-established B-cell and CLL TFs *NF-kB, AP2, P53, E2F1, PAX5* and *SP1* [71].

*SP1* is a ubiquitous transcriptional activator [72], whose binding to the DNA motif could be influenced by DNA methylation [73]. In humans, a number of genes with GC-rich promoter regions (*CiDEA* included) were found to be regulated by the combined effects of *SP1* and DNA methylation [70,73]. The association between *SP1* and DNA methylation has never been described before in *MAL* and *PCDH17*, despite the identification of *SP1*-binding sites in their promoters [74,75].

The steric interference of methylation at CG sites with TF binding to DNA has been described for *AP2* as well [76,77]. The activator protein AP2 binds a GC-rich DNA sequence motif discovered in the regulatory components of cancer-related central growth and differentiation genes reviewed in [76,78]. *AP2* and Zinc-finger TFs were shown to be among the differentially methylated genes in cDLBCL [13]. This result suggests that the methylation-mediated inhibitory effect on genes transcriptionally controlled by *AP2* might be due to *AP2* gene methylation or *AP2*-binding site methylation, as seen in cDLBCL.

Many lymphoid malignancies, including human and canine DLBCL, have increased NF-kB signalling [3,79,80]. Recent molecular research has revealed that methylation of the CpG dinucleotide next to kB sites changes the regulatory activity of *NF-kB* [81]. To the best of the authors’ knowledge, only *CiDEA* has shown direct evidence of *NF-kB* participation in transcriptional control among the target genes studied here [70].

*PAX5*, a B-cell immunomarker [82], operates as a nuclear TF that regulates gene transcription by recruiting chromatin-remodelling, histone-modifying, and basal TF complexes to its target genes [83]. Interestingly, as with *AP2*, the methylation of *PAX5* binding sites identified here was concurrent with the aberrant hypermethylation of *PAX* genes reported in cDLBCL [13] and other human malignancies [84].

Finally, the myeloid zinc finger TF *MZF1* regulates differentiation, proliferation and programmed cell death, and its abnormal expression may result in the formation of haematological malignancies [85]. The effect of methylation on *MZF1*-targeted DNA binding motifs has previously been described for the reprogramming key gene *OCT4* in induced pluripotent stem cells [86], the *PAX2* gene in endometrial cancer [87], and the tumour antigen *PRAME* (preferentially expressed antigen in melanoma) in melanoma cells [88]. There is no evidence that *MZF1* is involved in the regulation of *CiDEA, MAL* or *PCDH17*.

Overall, only a preliminary predictive analysis of *CiDEA, MAL* and *PCDH17* promoters was performed in this work. To validate our predictions and establish the participation of *AP2, MZF1, NF-kB, PAX5* and *SP1* TFs or other TFs not initially considered in the methylation-dependent regulation of *CiDEA, MAL* and *PCDH17* in cDLBCL, chromatin immunoprecipitation assays were required.

The similarities in the epigenetic mechanism of regulation between cDLBCL and other human cancers, as well as evidence of antitumourigenic activity of *CiDEA, MAL* and *PCDH17* after ectopic re-expression in other human cancers, led us to hypothesise that these three genes, whose expression was restored by epigenetic drugs, could represent new potential drivers in cDLBCL. This hypothesis paves the way for further research aiming at understanding the functional significance of these possible TSGs in this cDLBCL. Furthermore, additional characterisation and experiment-based validation of the discovered regulators (predicted TFBSs) may result in the finding of novel epigenetically dysregulated pathways in cDLBCL, providing new insights into the role of DNA methylation changes in cancer.

## 4. Materials and Methods

### 4.1. Chemicals and Reagents

Cell culture basal media (RPMI 1640, IMDM, DMEM and OPTI-MEM) and additives (foetal bovine serum, FBS; L-glutamine; non-essential amino acids; penicillin and streptomycin; sodium pyruvate) were all from Gibco, Life Technologies (Carlsbad, CA, USA). AZA, DEC, VAL, TSA and SAHA were purchased from Sigma-Aldrich (Milan, Italy). Stock solutions of DEC, TSA and SAHA were prepared in DMSO and stored at −20 °C. AZA and VAL were prepared in RPMI medium immediately before use. Anti-human MAL (E-1, sc390687) monoclonal antibody and goat anti-human glyceraldehyde-3-phosphate dehydrogenase (GAPDH, V-18, sc20357) polyclonal antibody were purchased from Santa Cruz Biotechnology (Dallas, TX, USA); rabbit anti-CiDEA (N1C3, GTX113166) and rabbit anti-PCDH17 (C-term, GTX45400) polyclonal antibodies were sourced from Genetex (Irvine, CA, USA). HRP-conjugated goat anti-rabbit (AP132P) and rabbit anti-goat (AP106P) IgG antibodies were obtained from Millipore (Burlington, MA, USA), while HRP-conjugated goat anti-mouse IgG antibody (GTX213111) was obtained from Genetex.

### 4.2. Candidate Genes Selection

Gene expression, DNA methylation and CNVs data obtained from the previous analysis of 50 cDLBCL samples and 11 control LNs were considered [7]. The protein coding genes significantly hypermethylated in cDLBCLs, specifically in the promoter (−1000 bp to TSS) and/or intergenic (−10,000 to −1000 bp from TSS) and/or exonic (the first exon, from TSS to +500 bp) regions, were crossed with the list of significantly downregulated or not expressed genes that were not subjected to gain or losses (lists 1, 2, 3 and 4, Figure S1). As per “not expressed” genes in the cDLBCL samples (list 4), we referred to those genes showing a normalized expression level above the threshold (10 reads) in the LN specimens only. A further list (list 5, Figure S1) included not redundant hypermethylated, downregulated and CNVs-free genes showing a significant (*p* < 0.05) inverse correlation between MET and GEX data. To assess the above-mentioned correlation, pairwise Pearson’s correlation coefficients were calculated for each gene using R software v3.1.3.

The preliminary set of candidate genes (lists 1, 2, 3, 4, 5) was then skimmed using a literature-based filtering approach. Briefly, the literature provided by Database for Annotation, Visualisation and Integrated Discovery (DAVID) v6.8 software and related to both human and canine gene IDs was considered; the priority was assigned to the following keywords: epigenetics, methylation, lymphoma, cancer, metastases, oncogene, tumour suppressor and silencing.

Finally, the candidate genes (*n* = 21, Figure S1) selected for the experimental filtering (Figure 1) were definitively chosen using the following selection criteria: a statistically significant inverse correlation between MET and GEX data; a mutually exclusive plotting of MET and GEX data between cDLBCL and LN samples at specific CpG sites (Figure S6). Additionally, since the following experimental filtering was partly executed in CLBL-1 cells, the hypermethylation and downregulation of candidate genes in CLBL-1 cells vs. LNs were verified. In this respect, data previously published by [7] were used.

### 4.3. Canine B-Cell Lymphoma Cell Lines

Both established and primary B-cell lymphoma cell lines were considered. CLBL-1 cells, isolated from the peripheral lymph node of a dog with confirmed stage IV cDLBCL [28], were grown in RPMI 1640 medium, supplemented with 10% FBS, 2 mM L-glutamine, 1% non-essential amino acids and 100 U/mL penicillin and 100 µg/mL streptomycin.

cDLBCL PCCs (*n* = 4) were obtained from fresh surgery-derived lymphoma tissues harvested for diagnostic purposes. The final diagnosis of cDLBCL was made according to the WHO classification of canine lymphoma [89], including morphologic and immunohistochemical criteria. After surgery, tissue specimens (~1 cm^3^) were reduced in small pieces, stored in cold PBS 1X + 10% FBS and transferred to the laboratory. The tissue was then crumbled in the same buffer and filtered in 40 μm cell strainers (Becton Dickinson, San Josè, CA, USA). Cells were centrifuged at 1700× *g* for 8 min and the pellet was resuspended in RPMI 1640 medium containing 2 mM L-glutamine, 10% FBS and 100 U/mL penicillin and 100 µg/mL streptomycin. Lymphocytes were then isolated through a gradient centrifugation (1200× *g* for 20 min) in Ficoll Histopaque^®^ 1077 (Thermo Fisher Scientific, Waltham, MA, USA), washed in a PBS solution and cultured in IMDM with 10% FBS, 2 mM L-glutamine, 100 U/mL penicillin and 100 µg/mL streptomycin, 1 mM sodium pyruvate and 1% non-essential amino acids. Cells were seeded in T25 or T75 flasks at a density of 2.5 × 10^6^ cells/mL. The cell number was assessed with CountessTM II Automated Cell Counter (Thermo Fisher Scientific, Waltham, MA, USA). PCCs were immunophenotyped through flow cytometry analysis (CyFlow^®^ Space, Sysmex^®^ GmbH, Norderstedt, Germany) using anti-CD45, CD4, CD5, CD8, CD21, CD25, CD34 antibodies as previously reported [90], and characterised by clonality assessment (PARR) following the method reported in [91]. Clinical data (signalment, clinical stage and substage) of the 4 dogs included in the current series as well as laboratory findings at isolation time (cell count, mortality estimated with propidium iodide staining, flow cytometry immunophenotyping and clonality assessment by PARR) are reported in Table S1. All applicable international and national guidelines for the care and the use of animals were followed, and dogs’ owners were required to give written informed consent for the use of sample biopsies for research purposes.

### 4.4. Cell Treatments

To analyse the effects of DNA methylation and/or histone acetylation on gene expression, CLBL-1 and PCC cells were treated with HDs and HDACis. Briefly, CLBL-1 cells were seeded at a concentration of 3 × 10^5^ cells/well in a 6-well flat-bottom plate (Sarstedt Italia, Verona, Italy), and incubated for 72 h with AZA (3.4 μM) or DEC (0.13 μM) alone or in combination with HDACis. Valproic acid (1.7 mM), SAHA (0.7 μM) or TSA (0.012 μM) were added in the last 24 h of incubation, as previously reported [24]. The final concentrations of HDs and HDACis here used corresponded to the previously determined IC_50_ and IC_20_ values, respectively. Four independent cultures were executed. In each experiment, cells treated with the vehicle only were included. PCCs (*n* = 4) were seeded at a density of 6 × 10^6^ cells/well in P6 multiwell plates and treated with HDs and HDACis following the same experimental protocol used for CLBL-1 cells. At the end of the experiment, cells were washed with PBS and collected for nucleic acid extraction (total RNA and genomic DNA).

### 4.5. Cytotoxicity

A cytotoxicity screening was performed to assess the availability of PCCs after the exposure to HDs and HDACis. PCCs were seeded in a 96-well flat-bottom plate (Sarstedt Italia, Verona, Italy) at a concentration of 4 × 10^5^ cells/well and incubated with AZA, DEC, VAL, SAHA and TSA using the same experimental conditions described above. Additional wells were exposed either to the vehicle (dimethyl sulfoxide, DMSO, 0.1% final concentration) for DEC, TSA and SAHA or to the cell culture medium (for AZA and VAL). Each treatment condition was tested in sextuplicate. At the end of the incubation time, cell viability was measured using CellTiter-Blue Reagent (Alamar Blue, Promega, Madison, WI, USA) as previously described [24], and expressed as a percentage relative to that of the respective control.

### 4.6. Quantitative Real-Time PCR (qPCR)

A candidate gene approach was used to evaluate the expression of selected target genes (Figure S1) in tissue specimens (cDLBCL and normal follicular B-cells), as well as in CLBL-1 and PCCs cells treated with HDs and HDACis. Total RNA was extracted from all samples using the RNeasy Mini Kit (Qiagen, Hilden, Germany) as per manufacturer’s instructions. Concentrations were measured with NanoDrop 1000 Spectrophotometer (Thermo Fisher Scientific, Waltham, MA, USA). An amount of 1 µg of total RNA was reverse transcribed using the High Capacity cDNA Reverse Transcription kit (Life Technologies, Carlsbad, CA, USA), according to the manufacturer’s instructions. For each target transcript, gene-specific primers that encompassed 1 intron were designed by using Primer3 software (http://primer3.ut.ee/, accessed on 1 July 2018). Oligonucleotides were synthesized by Eurofins MWG Synthesis GmbH (Ebersberg, Germany) and are reported in Table S4. The qPCR amplification was performed in duplicate in a Stratagene Mx3000P thermal cycler (Agilent Technologies, Santa Clara, CA, USA) in a final volume of 10 μL, using 12.5 ng of cDNA, the oligonucleotide concentration (range 300–600 nM) defined in the preliminary assay set up (Table S4) and 2X Power SYBR Green PCR Master Mix (Life Technologies, Carlsbad, CA, USA). Standard qPCR conditions were used. Two internal control genes (ICGs: *GOLGA1* and *CCZ1*) were considered [24]. Additionally in pharmacologically unmasking experiments, *RPL8* was included as negative control gene as it was not affected by HD and HDACi treatment [24]. Standard curves were obtained using the best performing primer combination and serial dilutions of cDNA from control LN. The ∆∆Ct method was used to analyse gene expression results. A total of 13 out of 21 qPCR assays had an acceptable efficiency (range 90% ÷ 110%), and a slope in the range of −3.6/−3.1 (Table S4).

### 4.7. Methyl Specific PCR (MSP)

Genomic DNA from CLBL-1 and PCC cells was extracted using the DNeasy Blood and Tissue Kit (Qiagen, Hilden, Germany) and quantified with NanoDrop 1000 Spectrophotometer. There was 500 ng of gDNA bisulfite-converted following the MethylCode™ Bisulfite Conversion Kit (Life Technologies, Carlsbad, CA, USA) as per the manufacturer’s instructions. For each gene (*CiDEA, MAL* and *PCDH17*), 2 couples of primers, 1 specific for the methylated DNA (Meth) and 1 for the unmethylated DNA (No Meth), were designed using Methyl Primer Express software v1.0 (Applied Biosystems, Foster City, CA, USA), as previously described [92,93]. The DNA sequence considered for the primer design was obtained from the alignment of *CiDEA*, *MAL* and *PCDH17* CpG-rich regions, identified by MBD-seq [7], to the canine genome. Among tested genes, *RPL8* was included as a negative control gene [24]. The list of Meth and No Meth primer pairs, as well as the concentration used, are reported in Table S5. The converted DNA (dilution 1:100) was processed in a final volume of 10 µL using the Power SYBR Green PCR Master Mix (Life Technologies, Carlsbad, CA, USA). The amplification was performed in a Stratagene Mx3000P thermal cycler (Agilent Technologies, Santa Clara, CA, USA using standard PCR conditions [24], except for *CiDEA*, for which a fluorescence signal acquisition temperature of 77 °C was set. The specific amplification was checked loading MSP products in a 2% agarose gel and analysing the melting curves. For each gene, the level of methylation was estimated by calculating the ratio of unmethylated to methylated assays as ΔCt (=Ct No Meth − Ct Meth), as previously described [24,94]. In case of absence of No Meth assay amplification, a Ct value of 40 was arbitrarily assigned to allow the ΔCt calculation.

### 4.8. Total Protein Isolation and Immunoblotting

CLBL-1 cells were seeded in 90 mm Petri dishes (Sarstedt Italia, Verona, Italy) at a concentration of 2.2 × 10^6^ cells and incubated with AZA or DEC alone and/or in combination with VAL, as described above. Six independent experiments were executed. After 72 h of incubation, cell pellets were solubilized in cold RIPA buffer (50 mM Tris-HCl, pH 7.4; 1% Triton X-100; 0,5% sodium-deoxycholate; 0,1% SDS; 150 mM NaCl; 2 mM EDTA; 1% protease inhibitor cocktail; all from Sigma-Aldrich, Milan, Italy), incubated on an ice bed for 30 min and centrifuged at 8000 rpm for 20 min. Total protein content was quantified using Pierce™ BCA Protein Assay Kit (Thermo Fisher Scientific, Waltham, MA, USA).

Whole protein lysates (15–30 µg) were separated in 4–12% SDS-polyacrylamide gels (NuPAGE Novex Bis-Tris Gels, Thermo Fisher Scientific, Waltham, MA, USA) using the XCell SureLock Mini-Cell electrophoresis system (Thermo Fisher Scientific, Waltham, MA, USA) and transferred onto nitrocellulose filters through the iBlot Dry Blotting System (Thermo Fisher Scientific, Waltham, MA, USA). On each gel, a prestained (PageRuler Plus Prestained Protein Ladder, Thermo Fisher Scientific, Waltham, MA, USA) and an unstained (MagicMark™ XP Western Protein Standard, Thermo Fisher Scientific, Waltham, MA, USA) molecular marker, as well as human and canine positive controls (see below) were loaded. After a blocking step for 2 h at 4 °C in Tris-buffered saline (TBS) buffer containing 0.05% Tween-20 and 5% powder milk, membranes were incubated first overnight at 4 °C with anti-human CiDEA, MAL, GAPDH and PCDH17 primary antibodies (dilution 1:1000; MAL 1:500) and then for 2 h with the appropriate HRP-conjugated secondary antibodies (dilution 1:5000). GAPDH was selected as reference protein (loading control) for the absence of effects due to HDs and HDACis exposure. The specific proteins were detected by the SuperSignal West Pico PLUS Chemiluminescent Substrate Kit (Thermo Fisher Scientific, Waltham, MA, USA) according to manufacturer’s instructions and the bands were automatically captured with iBright™ FL1000 Imaging System (Thermo Fisher Scientific, Waltham, MA, USA). Because of the low protein amounts, the blots developed with chromogenic substrates were stripped of antibodies using Restore^TM^ Western Blot Stripping Buffer (Thermo Fisher Scientific, Waltham, MA, USA) as per manufacturer’s instructions and reprobed. The raw Integrated Optical Density (IOD) of each band was acquired with ImageJ software (U.S. National Institute of Health, Bethesda, MD, USA). For the protein semi-quantification, the IOD of the specific bands of each sample was normalized firstly to the IOD of the loading control (GAPDH) and subsequently to the IOD of the canine positive control, selected as the calibrator (CF2Th_CiDEA for CiDEA, CF2Th_MAL for MAL and MDCK for PCDH17).

As per human and canine positive controls, total proteins isolated from HepG2, MCF7 and MDCK established cell lines as well as from Cf2Th cells transiently transfected with the full coding sequence of canine *CiDEA* and *MAL* were used. *CiDEA* (ENSCAFT00000036541) and *MAL* (ENSCAFT00000011303) full-length sequences were cloned into pCI-neo mammalian expression vector (Promega, Madison, WI, USA), as previously described [95]. The oligonucleotides used for the preliminary PCR amplification of total cDNA and for Nested-PCR are reported in Table S6. Ten different cDNA samples isolated from canine pathological and normal tissue biopsies were considered for the first PCR reaction. Cf2Th cells were finally transiently transfected (see paragraph 4.10 for details) with *CiDEA* or *MAL* plasmids, whose full sequence was previously confirmed by Sanger sequencing; 48 h post-transfection, cells were subjected to lysis and total proteins isolation.

### 4.9. Cloning of CiDEA, MAL and PCDH17 CpG-Rich Genomic Regions

Based on MBD-sequencing results [7], 2 CpG-rich regions for *CiDEA*, 1 for *MAL* and 5 for *PCDH17* were considered. Canine genomic DNA (30–50 ng) from the whole blood of 3 mixed-breed dogs was used to generate 8 long PCR fragments of ~1.0–1.5 Kbp. Amplicons were then purified and used as template for Nested-PCR to add restriction ends. In both amplification steps, the Q5 High-Fidelity DNA Polymerase (New England BioLabs, Ipswich, MA, USA) and Proflex thermal cycler (Thermo Fisher Scientific, Waltham, MA, USA) were used. Primer sequences and annealing temperatures used in both amplification steps are reported in Tables S7 and S8. Each Nested-PCR fragment and the pCpGL-basic vector (a CpG-free plasmid kindly donated by Maja Klung and Michael Rehli, University Hospital, Regensburg, Germany) were double-digested with two Fast Digest restriction enzymes among BamHI, BcuI, HindIII, NcoI and PstI (Thermo Fisher Scientific, Waltham, MA, USA) (see Table S8 for details) and ligated with T4 DNA Ligase (New England Biolabs, MA, USA). *E. coli* PIR1 cells (Invitrogen, Life Technologies, Carlsbad, CA, USA) were then used for the cloning and maintenance of the fragments of interest. After plasmid purification with QIAprep Spin Miniprep kit (Qiagen, Hilden, Germany), the orientation and sequence of the 8 CpG islands were verified by Sanger sequencing (BMR Genomics, Padua, Italy).

### 4.10. Promoter Reporter Assays

To monitor the contribution of the 8 CpG-rich regions to the regulation of gene transcription, luciferase reporter assays were executed. Cf2Th canine normal thymus cell line obtained from European Collection of Authenticated Cell Cultures (ECACC, Porton Down, UK, Ref No. 90110521) was used for the heterologous transfection. Cf2Th cells (passages 70–95) were cultivated in DMEM supplemented with 10% FBS, 2 mM L-glutamine, 1% non-essential amino acids and 100 U/mL penicillin and 100 µg/mL streptomycin. Before transfection, cells were seeded in white 96-well flat-bottom plates at a density of 10^5^ cells/well in OPTI-MEM supplemented with 5% FBS and cultured overnight to reach 50–70% confluence. About 24 h after seeding, cells were transfected with 200 ng of a pDNA mixture (pCpGL-basicΔ and pGL4.75[hRluc/CMV], ratio 4:1) using FuGene HD Transfection Reagent (Promega, Madison, WI, USA) at a FuGene:DNA ratio of 4:1. After 24 h the transfection was stopped, and cells were maintained in DMEM medium for a further 24 h before measuring luciferase activity with the Dual-Glo^®^ Luciferase Assay System kit (Promega, Madison, WI, USA) as per the manufacturer’s instructions. Renilla luciferase activity was used for the normalization of Firefly luciferase signal. The relative luciferase activity was finally expressed in –fold changes (arbitrary units, AU) as normalized to the negative control (empty pCpGL-basicΔ vector) to which an arbitrary value of 1 was assigned. Three independent experiments were performed, and each experimental condition was tested in sextuplicate. A relative luciferase activity value higher than 100 AU was arbitrarily chosen as the cut-off for the selection of the CpGI-rich regions subjected to in vitro methylation.

### 4.11. In Vitro Methylation

To obtain the direct evidence that the methylation in *CiDEA, MAL* and *PCDH17* promoter regions mediated the silencing of gene transcription, the pDNA constructs were subjected to in vitro methylation and dual luciferase reporter assays. The CpG-rich regions that mostly contributed to the activation of luciferase activity (at least one per gene) were selected. CpG methylation was performed by incubating 2 or 3 µg of each pDNA construct with SssI, HhaI and HpaII methyltransferases (New England Biolabs, Ipswich, MA, USA) alone or in combination (HhaI + HpaII) for 2 h at 37 °C. The methylation reaction was quenched by heating the solution at 65 °C for 20 min and finally verified by restriction enzyme digestion protection assay using methyl-sensitive HhaI and HpaII restriction endonucleases (New England Biolabs, Ipswich, MA, USA). Methylated plasmids were finally used for dual luciferase reporter assay in Cf2Th cells as described above. The luciferase activity of each methylated plasmid was expressed as the percentage of the respective unmethylated clone signal, to which an arbitrary value of 100% was assigned. Three independent experiments were performed, and each experimental condition was tested in sextuplicate.

### 4.12. In Silico Predictive Analysis

To predict the TFBSs located on *CiDEA, MAL* and *PCDH17* CpG-rich regions and potentially interested by DNA methylation, the outputs from 3 different tools were compared and integrated: MatInspector [96,97], Promo3 [98,99] and TFbind [100]. In the MatInspector tool the following parameters were set: *Homo sapiens* species, positive strand, matrix similarity score > 0.85, core similarity score > 0.85. In Promo3 the following settings were used: all factors’ species, human sites’ species, all matrices, dissimilarity values < 15%. In TFbind tool default settings were used.

### 4.13. Statistical Analysis

Results were expressed as the mean ± standard error of the mean (SEM). Statistical analysis was performed using GraphPad Prism 5 (San Diego, CA, USA). The unpaired T test or Mann Whitney test was used for comparison between 2 groups, while multiple group comparisons were conducted using one-way analysis of variance (ANOVA) followed by Bonferroni’s Multiple Comparison post-hoc test. A *p* < 0.05 was considered statistically significant.

## Data Availability

Data are contained within the article or Supplementary Material.

## Supplementary Materials

The following supporting information can be downloaded at: https://www.mdpi.com/article/10.3390/ijms23074021/s1.
