# Supplementary material for: Hypermethylation-Mediated Silencing of CIDEA, MAL and PCDH17 Tumour Suppressor Genes in Canine DLBCL: From Multi-Omics Analyses to Mechanistic Studies
Source: Int J Mol Sci. 2022 Apr 5;23(7):4021. doi: 10.3390/ijms23074021 (PMC9000013; doi:10.3390/ijms23074021)
Supplement: Supplementary file 1 [file ijms-23-04021-s001.zip › ijms-1653417-supplementary.pdf]

Article

Hypermethylation-mediated silencing of *CIDEA*, *MAL* and *PCDH17* tumour suppressor genes in canine DLBCL: from multi-omics analyses to mechanistic studies

Eleonora Zorzan [1], Ramy Elgendy [1,2], Giorgia Guerra [1], Silvia Da Ros [1], Maria Elena Gelain [1], Federico Bonsembiante [1,3], Giulia Garaffo [4], Nicoletta Vitale [4], Roberto Piva [4], Laura Marconato [5], Luca Aresu [6], Mauro Dacasto [1] and Mery Giantin [1,*]

[1] Department of Comparative Biomedicine and Food Science, University of Padua, 35020 Legnaro (PD), Italy; eleonora.zorzan@gmail.com (E.Z.); ramy.elgendy@astrazeneca.com (R.E.); giorgiaguerra94@gmail.com (G.Gu.); silviadaros31@gmail.com (S.D.R); mariaelena.gelain@unipd.it (M.E.G.); federico.bonsembiante@unipd.it (F.B.); mauro.dacasto@unipd.it (M.D.)

[2] Discovery Biology, Discovery Sciences, R&D, AstraZeneca, Gothenburg, Sweden; ramy.elgendy@astrazeneca.com (R.E.)

[3] Department of Animal Medicine, Productions and Health, University of Padua, 35020, Legnaro (PD), Italy; federico.bonsembiante@unipd.it (F.B.)

[4] Molecular Biotechnology Center, Department of Molecular Biotechnology and Health Sciences, University of Turin, 10126 Turin, Italy; giulia.garaffo@gmail.com (G.Ga.); nicoletta.vitale@unito.it (N.V.); roberto.piva@unito.it (R.P.)

[5] Department of Veterinary Medical Sciences, University of Bologna, 40064 Ozzano dell'Emilia (BO), Italy; laura.marconato@unibo.it

[6] Department of Veterinary Sciences, University of Turin, 10095 Grugliasco (TO), Italy; luca.aresu@unito.it

* Correspondence: mery.giantin@unipd.it; Tel.: +39.049.8272946

Abbreviations: αCGH, Array Comparative Genomic hybridization; ABC, activated B-cell like; ANOVA, one-way analysis of variance; AU, arbitrary units; AZA, azacytidine; BCL2, BCL2 apoptosis regulator; cDLBCL, canine diffuse large B-cell lymphoma; CIDE, DFF45-like effector; CiDEA, Cell-death-inducing DNA fragmentation factor α-like effector A; CLL, chronic lymphocytic leukemia; CNVs, copy number variations; CpG, cytosine-guanine sequence; CpGI, CpG island; CTRL, control; DAVID, Database for Annotation, Visualization and Integrated Discovery; DEC, decitabine; DFF45, DNA fragmentation factor-45; DLBCL, diffuse large B-cell lymphoma; DMSO, dimethyl sulphoxyde; ECACC, European Collection of Authenticated Cell Cultures; FBS, fetal bovine serum; GAPDH, glyceraldehyde-3-phosphate dehydrogenase; GEX, gene expression; HDACis, histone deacetylase inhibitors; HDs, hypomethylating drugs; ICGs, internal control genes; IOD, integrated optical density; LNs, lymph nodes; MAL, Myelin and Lymphocyte protein; MBD, Methyl-CpG-binding sequencing; MET, DNA methylation; Meth, methylated DNA; MSP, Methyl Specific PCR; MYC, MYC protooncogene; NF-kB, nuclear factor kB; No Meth, unmethylated DNA; PCCs, primary cell cultures; PCDH17, Protocadherin 17; PRAME, preferentially expressed antigen in melanoma; qPCR, quantitative Real Time PCR; RNA-seq, RNA sequencing; RQ, relative expression values; SAHA, vorinostat; SEM, standard error of the mean; TBS, Tris-buffered saline buffer; TFBSs, transcription factor binding sites; TFs, transcription factors; TSA, trichostatin A; TSGs, tumour suppressor genes; TSS, transcription starting site; VAL, valproic acid.

Tables

Table S1. Canine DLBCL primary cell cultures (PCCs): signalment and clinical stage of dogs included in the study as well as laboratory findings at isolation time.

Primary cell culture ID	Histological diagnosis	Signalment	Clinical stage and substage	Total cell number	Mortality (%)	Immunophenotyping	Clonality assessment
**PCC2**	DLBCL	Maltese, F, 13 yy	III a	$5.52*10^8$	18.0	CD21/CD45+ 71%, CD5+ 13%, CD4+ 6.4%, CD8+ 5%	IgH +, TCRγ -
**PCC5**	DLBCL	Crossbred, F, 10 yy	V b	$7.88*10^8$	2.7	CD21+ 66.2%, CD5+ 2.5%, CD4+ 1.7%, CD8+ 0.2%, CD25+ 16.7%, CD34-	IgH +, TCRγ -
**PCC6**	DLBCL	Dobermann, F, 6 yy	V a	$8.04*10^8$	2.5	CD21+ 74%, CD5+ 2.5%, CD4+ 1.7%, CD8+ 0.8%, CD25+ 17.7%, CD34-	IgH +, TCRγ -
**PCC7**	DLBCL	Beagle, M, 6 yy	V b	$1.17*10^9$	4.0	CD45+ 66%, CD21+ 54%, CD5+ 4%	IgH +, TCRγ -

Table S2. Cytotoxicity of hypomethylating agents (AZA and DEC) and histone deacetylase inhibitors (VAL, SAHA and TSA) in cDLBCL primary cell cultures (PCCs).

Cell lines	Cytotoxicity (%)*				
	**AZA 3.4 μM**	**DEC 0.13 μM**	**VAL 1.7 mM**	**SAHA 0.7 μM**	**TSA 0.012 μM**
**PCC2**	n.a.	n.a.	n.a.	n.a.	n.a.
**PCC5**	51.8	0.0	35.6	17.3	11.9
**PCC6**	59.4	10.0	41.1	22.2	24.6
**PCC7**	74.0	2.0	16.6	12.3	32.4

* Citotoxicity vs. the respective control (medium for AZA and VAL; DMSO for DEC, SAHA and TSA); n.a.: not available. Alamar blue test was used.

Table S3. Putative transcription factors binding sites predicted in CiDEA_CpGI1, MAL_CpGI1, PCDH17_CpGI1 and PCDH17_CpGI3 regions.

TFs	DESCRIPTION	MATRIX FAMILY	CiDEA_CpGI1	MAL_CpGI1	PCDH17_CpGI1	PCDH17_CpGI3
**AP1**	AP-1 Transcription Factor	V$AP1R		X		
**AP2**	AP-2 Transcription Factor	V$AP2F	X	X	X	X
**AP4**	AP-4 Transcription Factor	V$AP4R	X			X
**CMYB**	MYB Proto-Oncogene, Transcription Factor	V$MYBL			X	X
**CREB1**	cAMP Responsive Element Binding Protein 1	V$CREB				X
**EBF1**	Early B-Cell Factor 1	V$EBF1	X	X		
**EGR1**	Early Growth Response 1	V$EGRF	X		X	
**EGR2**	Early Growth Response 2	V$EGRF			X	
**ERα**	Estrogen Receptor Alpha	V$ER	X			
**GATA1**	GATA-Binding Factor 1	V$GATA				X
**GCF2**	GC-Rich Sequence DNA-Binding Factor 2	V$GCF2	X			
**GFI1**	Growth Factor Independent 1 Transcriptional Repressor	V$GFI1				X
**HINF-P**	Histone H4 Transcription Factor	V$HNFP		X		
**HSF2**	Heat Shock Transcription Factor 2	V$HEAT				X
**LYF1**	Lymphoid Transcription Factor 1	V$IKRS			X	X
**MECOM**	Zinc Finger Protein Evi1	V$EVI1		X		
**MYOD1**	Myogenic Differentiation 1	V$MYOD				X
**MZF1**	Myeloid Zinc Finger 1	V$MZF1	X	X	X	X
**NF1**	Neurofibromin 1	V$NF1F			X	X
**NF-kB**	Nuclear Factor-kappa B	V$NFKB	X	X	X	X
**NKX2-5 (HMX2)**	NK2 Homeobox 5	V$NKXH		X		X
**P300**	P300 coactivator	V$P300				X
**P53**	P53 Tumor Suppressor	V$P53F		X		
**PATZ1**	POZ/BTB and AT Hook Containing Zinc Finger 1	V$MAZF	X	X		
**PAX5**	Paired Box Gene 5 (B-cell Lineage Specific Activator)	V$PAX5	X	X	X	X
**RFX1**	Regulatory Factor X1	V$XBBF		X		X
**SP1**	Stimulating Protein 1, ubiquitous zinc finger transcription factor	V$SP1F	X	X	X	X
**SPI-1**	Hematopoietic Transcription Factor PU.1	V$ETSF		X		
**STAT1**	Signal Transducer and Activator of Transcription 1	V$STAT		X		
**STAT3**	Signal Transducer and Activator of Transcription 3	V$STAT		X		
**TAL1**	TAL BHLH Transcription Factor 1, Erythroid Differentiation Factor	V$HAND		X		X
**TBX2 (T gene)**	T-Box Transcription Factor 2	V$BRAC		X		
**WT1**	WT1 Transcription Factor	V$EGRF		X	X	
**ZBTB7A**	Zinc Finger and BTB Domain Containing 7A	V$ZF02		X		X
**ZNF02 (ZNF219)**	C2H2 Zinc Finger Transcription Factors 2	V$ZF02		X	X	X

Table S4. Efficient qPCR assays used for mRNA amplification: oligonucleotides and assay parameters (efficiency %, R^2 and dynamic range).

Transcript	Ensembl ID	Primer sequence (5′ – 3′)	Amplicon length (bp)	Primer final concentration (nM)	Efficiency (%)	R^2	Dynamic range (Ct)
BMP7	ENSCAFT0000009354	F: TGGTCATGAGCTTCGTCAAC<br>R: AGCACCTGGTAAACGCTGAT	199	300/300	107	0.98	29.19 - 36.45
CD1D	ENSCAFT00000018156	F: TTCCAAGGGTCTCACTGGGTG<br>R: GGTCGCTCTTGCTTCTCCAGTT	185	300/300	105	1.00	27.15 - 33.23
CiDEA	ENSCAFT00000036541	F: ACGTGAAGGCCACCATGTAT<br>R: TGAGCAACTGTCCAGTCACC	129	300/50	95	0.99	30.40 - 36.20
CYP1A1	ENSCAFT00000028474	F: GCTTCATGCAAAAGATGGTCAAG<br>R: GTTGTGACTGTGTCAAATCCAGC	193	300/300	110	0.89	29.29 - 33.30
CXCL14	ENSCAFT00000001652	F: AAGTGCAAGTGCTCCCGAAA<br>R: GACACGCTCTTGGTGGTGAT	122	300/300	96	1.00	19.68 - 29.45
LEF1	ENSCATG00000017891	F: ACGAGTCCGAAATCATCCCG<br>R: TGCTTTCCGTCATCAGGGTG	112	300/50	109	1.00	21.80 - 27.38
LHX8	ENSCAFT00000043981	F: CCAAAACCAGCAAAAAGAGC<br>R: TGGCGTGCTCTACAATTCTG	176	300/300	93	0.99	22.88 - 33.60
MAL	ENSCAFT00000011303	F: TTCTCCGTCTTCACGACCTT<br>R: ACGAGGCAATCAAGATCCAC	85	300/50	93	1.00	19.22 - 31.09
PCDH17	ENSCAFT00000007840	F: CTGCCACTCGGATGTCCATA<br>R: CCTTAAACGTGGAGCTACTTTG	102	300/300	103	0.99	23.41 - 32.73
RIPK4	ENSCAFT00000016214	F: CAAGATCTGGACACGAAGCA<br>R: CTGAGAGGTACCCGAATCCA	144	50/50	110	0.92	32.84- 38.65
SCN3B	ENSCAFT00000018312	F: AGTTTGCTTTTGAAGCACATCG<br>R: TTCAGCCTTTGAGACCTTCCTG	191	300/50	97	0.99	22.89 - 34.02
SLC44A3	ENSCAFT00000032001	F: GTCGACATCCTCCATCGAAT<br>R: AGAAGGGTGGTGATGAATCG	128	50/50	105	0.91	29.79 - 37.38
TCF7	ENSCAFT00000001527	F: AGCACCAGGAATCTACCACAG<br>R: GCACTGTCATCGGAAGGAA	96	300/300	100	1.00	19.57 - 27.65
RPL8	ENSCAFT00000002627	Da Ros et al. (2018)*					
CCZ1	ENSCAFT00030034933	Da Ros et al. (2018)*			102	1.0	20.85 - 31.46
GOLGA1	ENSCAFT00000088944	Da Ros et al. (2018)*			102	1.0	23.30 – 33.71

*Da Ros, S.; Aresu, L.; Ferraresso, S.; Zorzan, E.; Gaudio, E.; Bertoni, F.; Dacasto, M.; Giantin M. Validation of epigenetic mechanisms regulating gene expression in canine B-cell lymphoma: An in vitro and in vivo approach. *PLoS One*. **2018**, *13*, e0208709. DOI: 10.1371/journal.pone.0208709.

Table S5. Oligonucleotides used for Methyl Specific PCR: sequence, final concentration and temperature set for the acquisition of the fluorescence signal.

Gene	Meth primers			No Meth primers		
	**Sequence (5′ – 3′)**	**[F/R] (nM)**	**T* (°C)**	**Sequence (5′ – 3′)**	**[F/R] (nM)**	**T* (°C)**
CiDEA	F: TATTTCGTTTCGGGGAAGC<br>R: AACACCCAACGAAAAACTCG	300/50	77	F: TATTTTGTTTTGGGGAAGTGT<br>R: CAACACCCAACAAAAAACTCA	300/50	77
MAL	F: GGTATGGTTTTAGTAGCGGC<br>R: CCCGTACAACCTACGACTC	50/50	60	F: GTATGGTTTTAGTAGTGGTGTT<br>R: CCATACAACCTACAACTCCA	50/50	60
PCDH17	F: ATATCGTAAACGTTGATTTGGC<br>R: CAACGCAAAAAACTAATATCCCG	300/300	60	F: TTTATATTGTAAATGTTGATTTGGT<br>R: ACAACACAAAAAACTAATATCCCA	300/300	60
RPL8	Da Ros et al. (2018)*	50/50	77	Da Ros et al. (2018)*	50/300	60

[F/R]: concentration of forward and reverse primers; * Temperature set for fluorescence signal acquisition

*Da Ros, S.; Aresu, L.; Ferraresso, S.; Zorzan, E.; Gaudio, E.; Bertoni, F.; Dacasto, M.; Giantin M. Validation of epigenetic mechanisms regulating gene expression in canine B-cell lymphoma: An in vitro and in vivo approach. *PLoS One*. **2018**, *13*, e0208709. DOI: 10.1371/journal.pone.0208709.

Table S6. Primer sequences and restriction enzymes used for *CiDEA* and *MAL* full-length sequence amplification and cloning.

	Primer sequence (5′-3′)	T used for PCR amplification (°C)	Amplicon length (bp)	Restriction enzymes
**CiDEA_#PCR1**	F: GGCGGGGCTTTAAGAGCG	60	786	-
	R: TATCTCCAAAATCATCATTTGAGAGGG			
**MAL_#PCR1**	F: AGCCGCGCGTCCAGCCCG	70	541	-
	R: GGAAGGCAGGCTGATCAGTTAACACCGTCT			
**CiDEA_#PCR2**	F: GAG**ctc**GA**g**CCA**a**C**c**ATGGAGACCGC	68	688	XhoI
	R: CCCTG**ac**GC**g**TGCCTATCTGCATGT			MluI
**MAL_#PCR2**	F: GTCCAGC**ta**GCC**ac**CATGGCCCCA	68	490	NheI
	R: AAGT**a**C**gcg**TGCTTTATGAAGACTTCCATCTGATT			MluI

#PCR1 from total cDNA; #PCR2 Nested-PCR.

The melting temperature recommended for Q5 High-Fidelity DNA Polymerase was calculated using a specific calculator freely available at http://tmcalculator.neb.com/. Sites recognized by XhoI, MluI and NheI restriction enzymes are highlighted in grey, while the Kozak sequence (5′-ACCATGG-3′) is underlined; bases that were modified respect to the wild type sequence are bolded and in lowercase.

Table S7. Primer pairs used to amplify *CiDEA*, *MAL* and *PCDH17* CpG-rich regions from canine genome and annealing temperature used.

GENE	CpG island position from TSS (+1)	CpG island length (bp)	Primer sequence (5'-3')	T used for PCR amplification
**CiDEA_CpG1**	+210/+510	300	F: GCGACACATCTACCTTTTCA<br>R: AGGCGTGGCCGCGAG	62°C
**CiDEA_CpG2**	+710/+1510	800	F: CCCCTCGTGCCCCCAGCG<br>R: GATCTTCAAAGAAGCACTGGCCCTTCC	65°C
**MAL_CpG1**	-822/+578	300	F: AAGATTAAATCTTAGTCCAGGAAGAAA<br>R: AGCTGGGCACTCCTACAAG	60°C
**PCDH17_CpG1**	-3779/-2779	1000	F: TGCTTCTGGAGGAAGACGGT<br>R: CTCTTCTATCACGGGTGGTCTC	60°C
**PCDH17_CpG2**	-2379/-1879	500	F: CCTCCAAAACATGAATCTGGCTTTT<br>R: GTAGGAGTGGGCTGGATGGAA	60°C
**PCDH17_CpG3**	-1679/-579	1100	F: CCATGAGTTGCCTGTTGATTTCC<br>R: GACTTGGGGACACTCAAAGTTGA	65°C
**PCDH17_CpG4**	-479/+721	1202	F: GGTTCGGGGAACTTGAGCGGAA<br>R: AGGACCCTGCACTGCAGCTG	60°C
**PCDH17_CpG5**	+821/+2421	1600	F: CAAGCTGGAGGAGAACTACGACAA<br>R: TCCTGGTCCGAAGGTGTCAGG	60°C

Positions of the amplified regions are relative to the transcription starting site (TSS, +1) of the respective gene. The melting temperature (Tm) recommended for Q5 High-Fidelity DNA Polymerase was calculated using a specific calculator freely available at http://tmcalculator.neb.com/.

Table S8. Oligonucleotides and restriction enzymes used for Nested-PCR and cloning of *CiDEA*, *MAL* and *PCDH17* CpG-rich regions.

GENE	Primer sequence (5'-3')	T used for PCR amplification	Restriction enzymes
**CiDEA_CpGI1**	F: GTC**a**C**t**AGTCACCCCGTCTCGG	65°C	BcuI
	R: GCA**a**GC**t**TGGCCGCGAGCC		HindIII
**CiDEA_CpGI2**	F: CTCCTGCA**g**CCGGCATCCC	60°C	PstI
	R: CTTCCA**t**G**g**GATATTTTATCAACAAAGACA		NcoI
**MAL_CpGI1**	F: AAACA**gg**ATCCAGGCAATGGGAGGG	65°C	BamHI
	R: CAAGG**c**C**a**T**g**GCCCTCTCGGAAGG		NcoI
**PCDH17_CpGI1**	F: GGTAGCTG**c**AGACTCTGGAGAGC	65°C	PstI
	R: TCAGC**c**CAT**g**GCCTGCAAAAGAC		NcoI
**PCDH17_CpGI2**	F: CAGAGAA**cta**GTTAGGAAGAAGCCG	60°C	BcuI
	R: GTGA**a**GC**t**TTCTGCAGTCTGGC		HindIII
**PCDH17_CpGI3**	F: AAACTGCA**g**CGAGAGGAGTCA	62°C	PstI
	R: GTGTCC**at**GGCTCCCGCTAG		NcoI
**PCDH17_CpGI4**	F: AAGGA**t**CCACGGACTCGGGGC	65°C	BamHI
	R: CCC**a**TGGCGCACGGAGACG		NcoI
**PCDH17_CpGI5**	F: CAGGAC**t**AGTACAACGTGACGA	61°C	BcuI
	R: AGTTA**a**GCT**t**CAAGTCTCTTACCTG		HindIII

Sites recognized by BcuI, HindIII, PstI, NcoI and BamHI restriction enzymes are highlighted in grey; bases that were modified respect to the wild type sequence are bolded and in lowercase. The melting temperature recommended for Q5 High-Fidelity DNA Polymerase was calculated using a specific calculator freely available at http://tmcalculator.neb.com/.

Figures

	MBD-seq			RNA-seq		aCGH	Statistical correlation	Number of not redundant genes		
	Hypermethylated									
	promoter	intergenic	exonic	Downregulated	Not expressed	no CNVs	MET vs GEX	First screening	Literature-based filtering	Selection
List 1	✓			✓		✓		162	24	11
List 2		✓		✓		✓		36	6	3
List 3			✓	✓		✓		44	6	4
List 4	✓	✓	✓		✓	✓		67	10	2
List 5	✓	✓	✓	✓		✓	✓	30	5	1
							Total no. of genes	339	50	21

Gene	Ensembl ID	Description	Category (list)
AJAP1	ENSCAFG00000019507	adherens junctions associated protein 1	HyperM*(promoter)_downregulated_noCNV**
BCL11B	ENSCAFG00000017809	B-cell CLL/lymphoma 11B	Correlated_HyperM_downregulated_noCNV
BMP7	ENSCAFG00000012011	bone morphogenetic protein 7	HyperM(promoter)_downregulated_noCNV
CD1D	ENSCAFG00000030554	CD1d molecule	HyperM(intergenic)_downregulated_noCNV
CiDEA	ENSCAFG00000018828	cell death-inducing DFFA-like effector a	HyperM(exonic)_downregulated_noCNV
CLDN3	ENSCAFG00000012532	claudin-3	HyperM(exonic)_downregulated_noCNV
CXCL14	ENSCAFG00000001084	C-X-C motif chemokine ligand 14	HyperM(promoter)_downregulated_noCNV
CYP1A1	ENSCAFG00000017937	cytochrome P450 family 1 subfamily A member 1	HyperM(promoter)_downregulated_noCNV
HOXA11	ENSCAFG00000002966	homeobox A11	HyperM_notexpressed_noCNV
HOXD10	ENSCAFG00000013447	homeobox D10	HyperM_notexpressed_noCNV
LEF1	ENSCAFG00000011252	lymphoid enhancer binding factor 1	HyperM(promoter)_downregulated_noCNV
LHX8	ENSCAFG00000020401	LIM homeobox 8	HyperM(exonic)_downregulated_noCNV
MAL	ENSCAFG00000007047	mal, T-cell differentiation protein	HyperM(promoter)_downregulated_noCNV
PAK5	ENSCAFG00000005685	p21 (RAC1) activated kinase 5	HyperM(promoter)_downregulated_noCNV
PCDH10	ENSCAFG00000003785	protocadherin 10	HyperM(exonic)_downregulated_noCNV
PCDH17	ENSCAFG00000004870	protocadherin 17	HyperM(intergenic)_downregulated_noCNV
RIPK4	ENSCAFG00000010213	receptor interacting serine/threonine kinase 4	HyperM(promoter)_downregulated_noCNV
SCN3B	ENSCAFG00000011538	sodium voltage-gated channel beta subunit 3	HyperM(promoter)_downregulated_noCNV
SLC44A3	ENSCAFG00000020108	solute carrier family 44 member 3	HyperM(promoter)_downregulated_noCNV
TCF7	ENSCAFG00000000990	transcription factor 7 (T-cell specific, HMG-box)	HyperM(intergenic)_downregulated_noCNV
TEKT3	ENSCAFG00000017923	tektin 3	HyperM(promoter)_downregulated_noCNV

*HyperM: hypermethylated gene

**noCNV: gene without Copy Number Variations

Figure S1. Candidate genes selection: summary of the bioinformatics filters used and list of selected targets. The number of genes identified in each step of the analysis is reported. Ensembl Genome Browser ID, description and category (list) is also provided. MBD-seq: Methyl-CpG-binding sequencing; CNVs: Copy Number Variations; MET: DNA methylation; GEX: gene expression

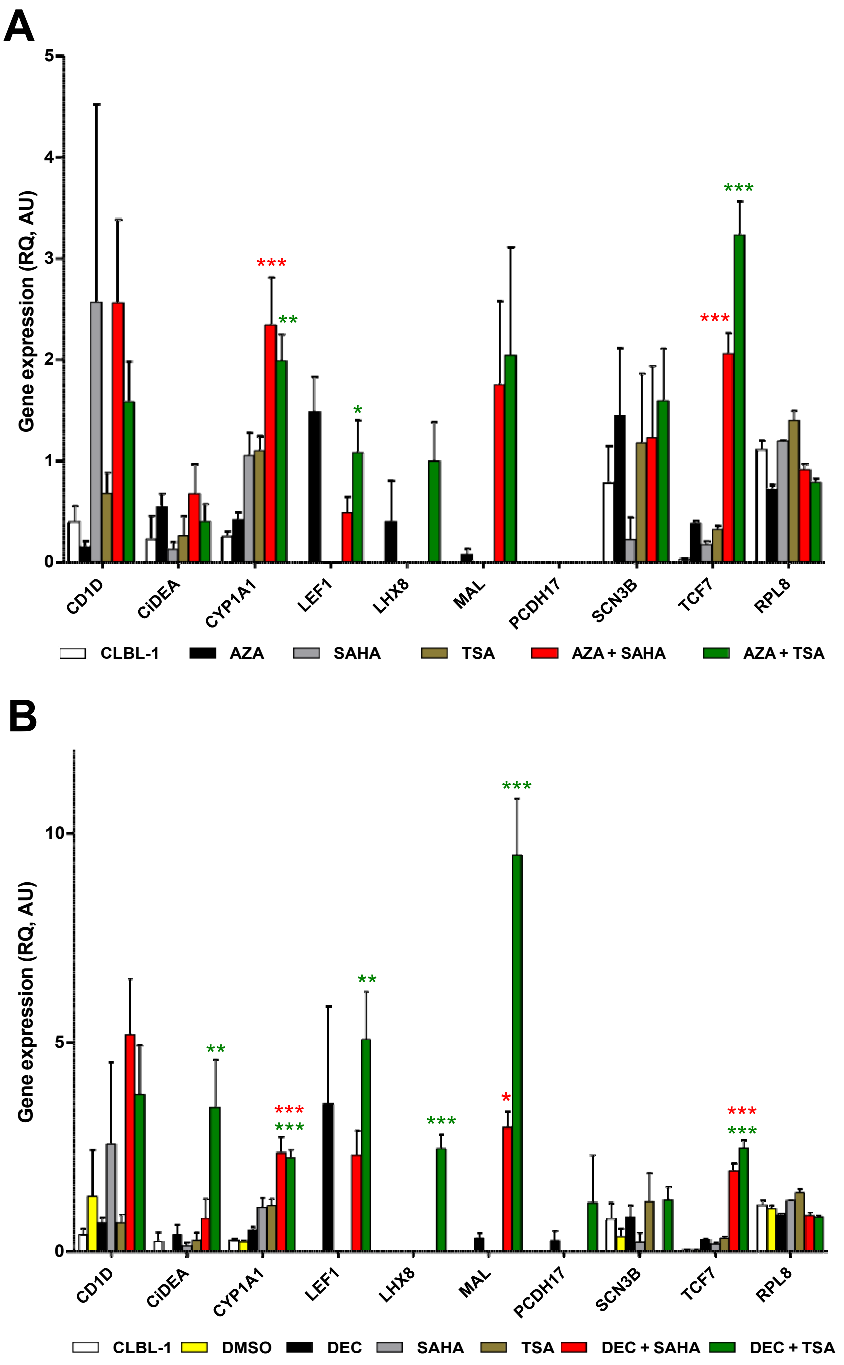

Figure S2. Effect of the treatment with AZA or DEC in combination with SAHA or TSA on the mRNA expression of 9 candidate TSGs in CLBL-1 cells. (A) AZA, (B) DEC. The expression levels of the target mRNAs (relative expression values, RQ), evaluated by qPCR and normalized to *GOLGA1* and *CCZ1*, are expressed in arbitrary units (AU), as the mean ± SEM of 4 independent experiments. *CXCL14, RIPK4* and *SLC44A3* are not shown because they were not expressed in CLBL-1 cells both in control and treatment conditions. *RPL8*, the negative control gene, was not affected by the treatment, as expected. Statistical analysis: ANOVA + Bonferroni post hoc test. *: $P < 0.05$; **: $P < 0.01$; ***: $P < 0.001$. Only the statistical significance between CLBL-1 vs AZA+SAHA or AZA+TSA and DMSO vs DEC+SAHA or DEC+TSA is shown.

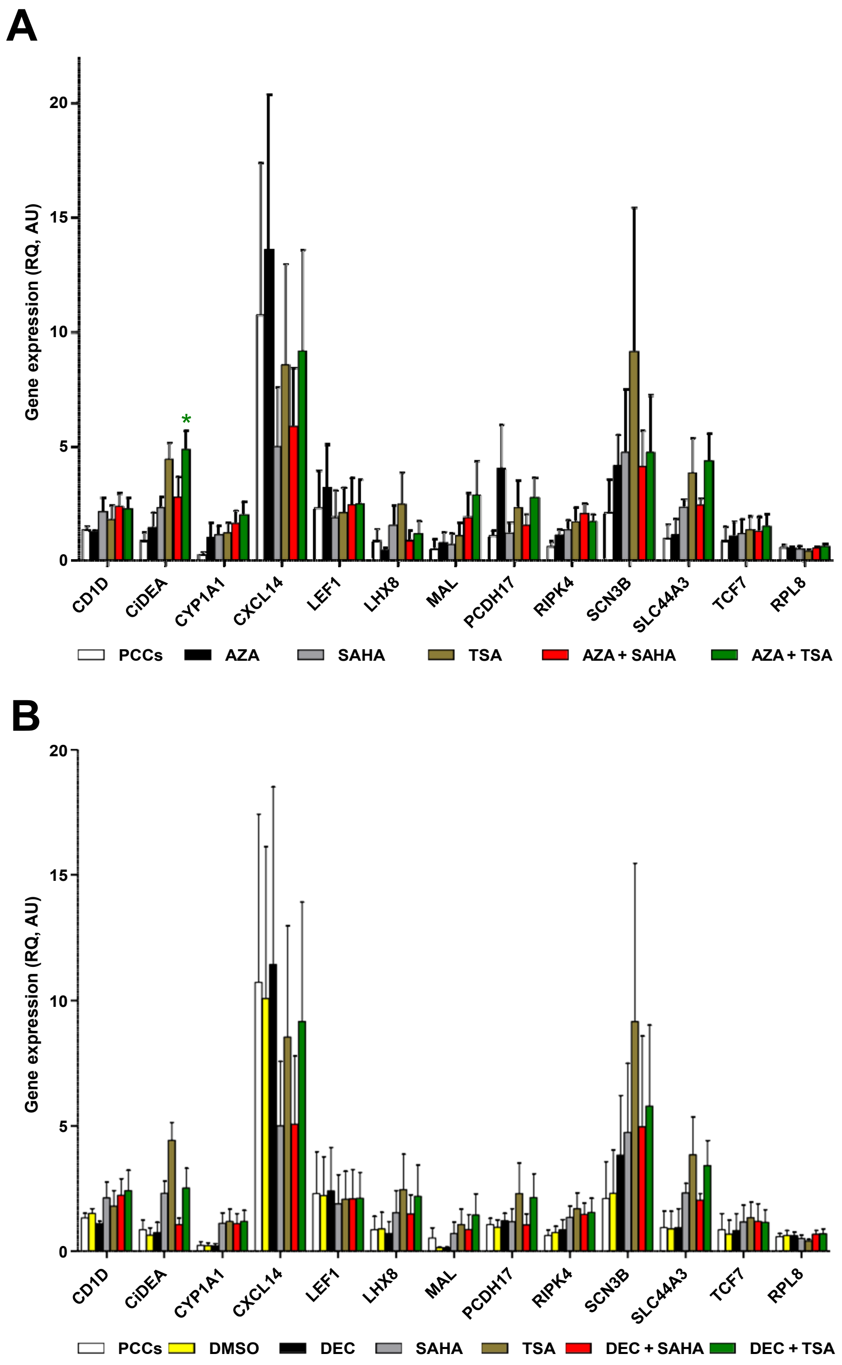

Figure S3. Effect of the treatment with AZA or DEC in combination with SAHA or TSA on the mRNA expression of 12 candidate TSGs in 4 cDLBCL primary cell cultures (PCCs). (A) AZA, (B) DEC. The expression levels of the target mRNAs (relative expression values, RQ), evaluated by qPCR and normalized to *GOLGA1* and *CCZ1*, are expressed in arbitrary units (AU), as the mean ± SEM. *RPL8*, the negative control gene, was not affected by the treatment, as expected. Statistical analysis: ANOVA + Bonferroni post hoc test. *: $P < 0.05$. The statistical significance between PCC vs AZA+SAHA or AZA+TSA and DMSO vs DEC+SAHA or DEC+TSA only is shown.

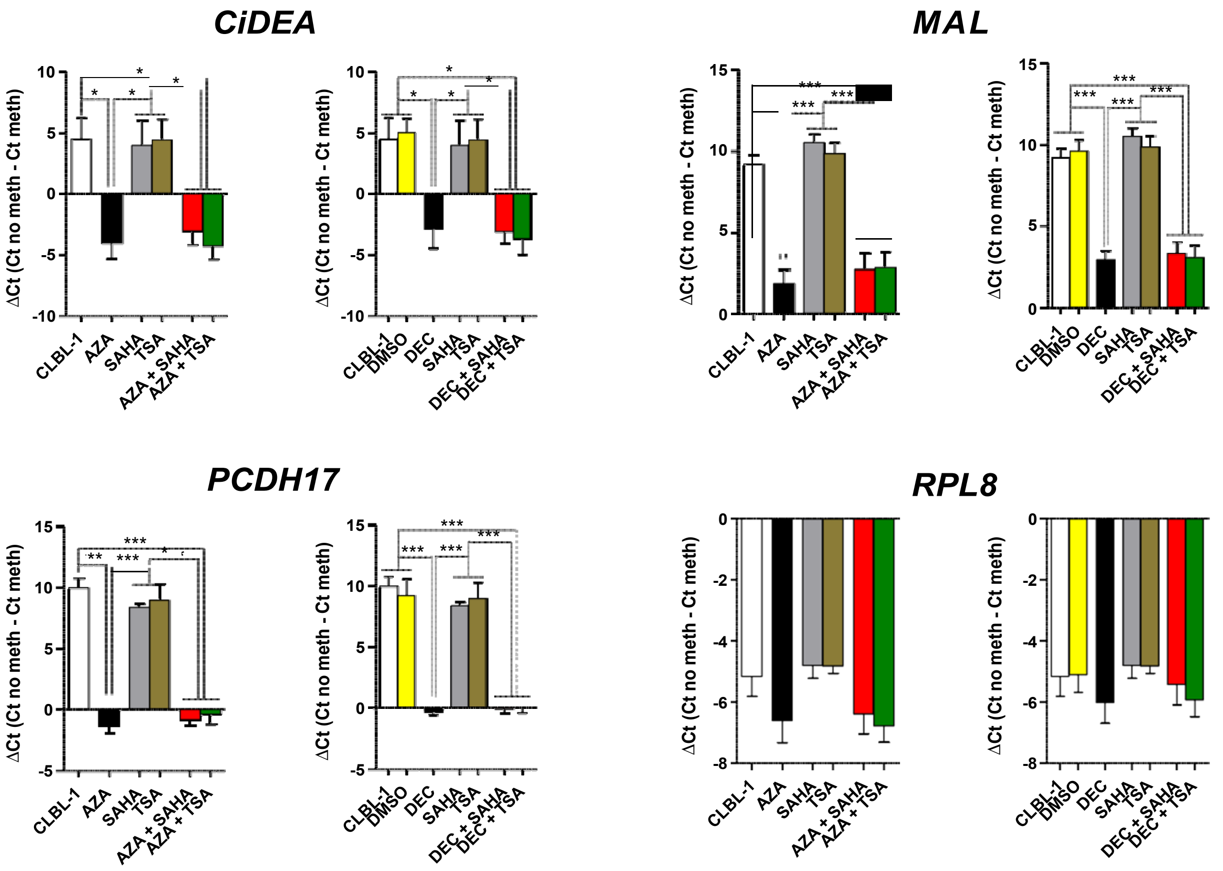

Figure S4. Effect of the treatment with AZA or DEC in combination with SAHA or TSA on *CiDEA, MAL, PCDH17* and *RPL8* methylation status in CLBL-1 cells. For each gene, the results of AZA are in the graph on the left, while results of DEC in the graph on the right. Data are expressed as ΔCt (= Ct No meth - Ct meth), as the mean ± SEM of 4 independent experiments. Four independent PLs were analysed. Statistical analysis: ANOVA + Bonferroni post hoc test. (*: $P < 0.05$; **: $P < 0.01$; ***: $P < 0.001$). *RPL8* (the negative control gene) was not affected by the treatment as expected.

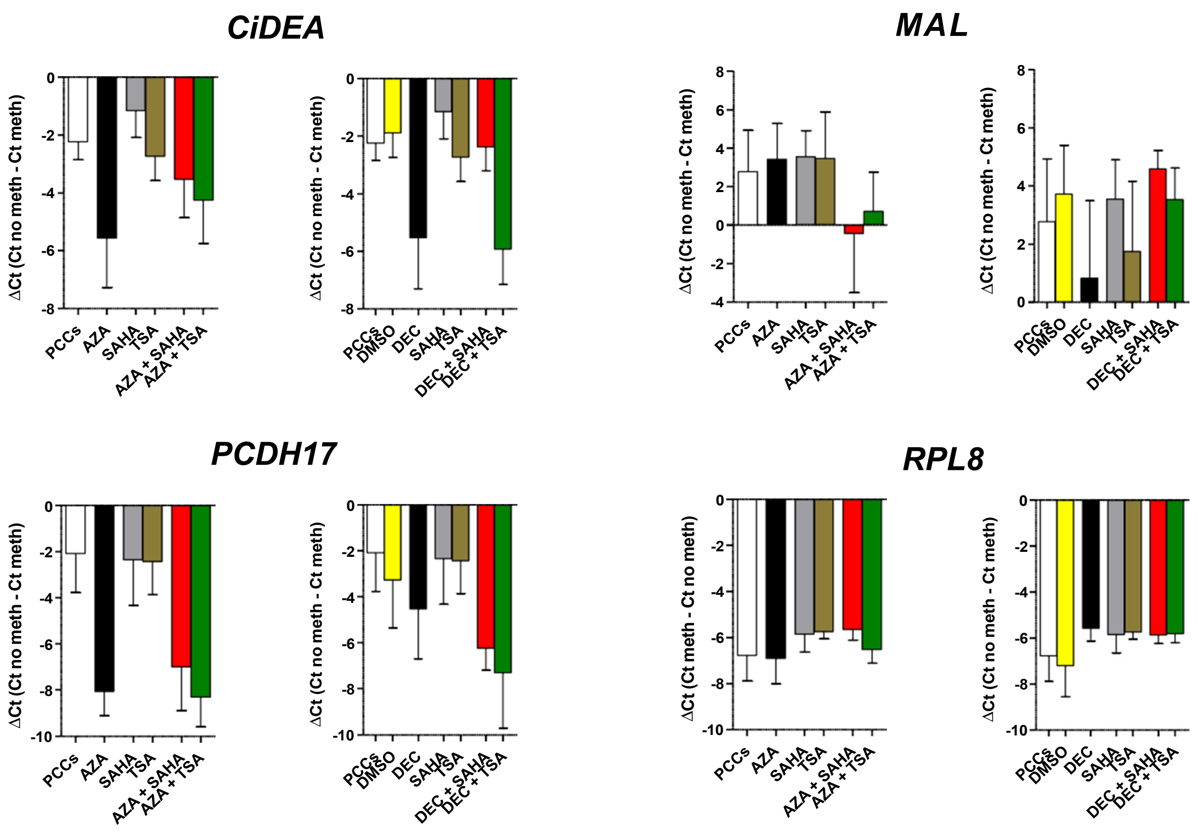

Figure S5. Effect of the treatment with AZA or DEC in combination with SAHA or TSA on *CiDEA, MAL, PCDH17* and *RPL8* methylation status in 4 cDLBCL primary cell cultures (PCCs). For each gene, the results of AZA are in the graph on the left, while results of DEC in the graph on the right. Data are expressed as ΔCt (= Ct No meth - Ct meth), as the mean ± SEM. Statistical analysis: ANOVA + Bonferroni post hoc test. (*: $P < 0.05$; **: $P < 0.01$; ***: $P < 0.001$). *RPL8* (the negative control gene) was not affected by the treatment as expected.

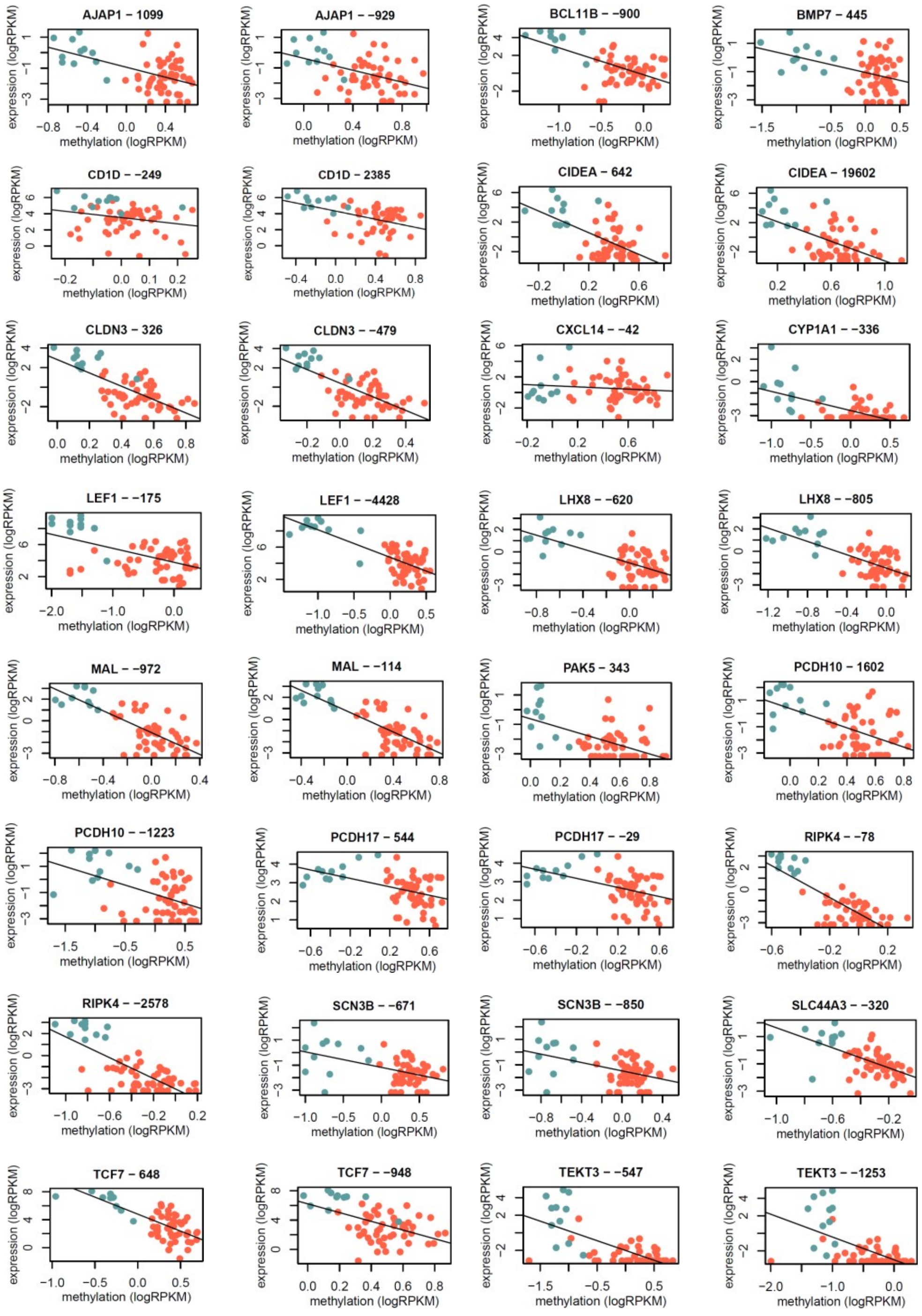

Figure S6. Correlations between DNA methylation and gene expression levels of candidate tumour suppressor genes in DLBCL and LN samples. Correlation charts for the complete cohort of samples (50 DLBCLs and 11 LNs) showing the inverse correlation ($P < 0.05$, $-1 < \text{rho} < 0$) between DNA methylation and gene expression data (expressed in logRPKM). The results of 19 out of 21 candidate tumor suppressor genes are reported (*HOXA11* and *HOXD10* were excluded because they were not expressed in DLBCL samples). Red and green dots indicate single DLBCL and LN samples, respectively. The name of the gene and the CpG-rich region position from TSS are reported on the top of each plot. Statistical analysis: pairwise Pearson's correlation.
